# FusionQ: a novel approach for gene fusion detection and quantification from paired-end RNA-Seq

**DOI:** 10.1186/1471-2105-14-193

**Published:** 2013-06-15

**Authors:** Chenglin Liu, Jinwen Ma, ChungChe (Jeff) Chang, Xiaobo Zhou

**Affiliations:** 1 Department of Diagnostic Radiology, Wake Forest School of Medicine, Winston-Salem, NC 27157, USA; 2 Department of Information Science, School of Mathematical Sciences and LMAM, Peking University, Beijing 100871, China; 3 Department of Pathology Florida Hospital, University of Central Florida, Orlando, FI 32803, USA

**Keywords:** Fusion detection, chimerical transcripts quantification, EM algorithm

## Abstract

**Background:**

Gene fusions, which result from abnormal chromosome rearrangements, are a pathogenic factor in cancer development. The emerging RNA-Seq technology enables us to detect gene fusions and profile their features.

**Results:**

In this paper, we proposed a novel fusion detection tool, FusionQ, based on paired-end RNA-Seq data. This tool can detect gene fusions, construct the structures of chimerical transcripts, and estimate their abundances. To confirm the read alignment on both sides of a fusion point, we employed a new approach, “residual sequence extension”, which extended the short segments of the reads by aggregating their overlapping reads. We also proposed a list of filters to control the false-positive rate. In addition, we estimated fusion abundance using the Expectation-Maximization algorithm with sparse optimization, and further adopted it to improve the detection accuracy of the fusion transcripts. Simulation was performed by FusionQ and another two stated-of-art fusion detection tools. FusionQ exceeded the other two in both sensitivity and specificity, especially in low coverage fusion detection. Using paired-end RNA-Seq data from breast cancer cell lines, FusionQ detected both the previously reported and new fusions. FusionQ reported the structures of these fusions and provided their expressions. Some highly expressed fusion genes detected by FusionQ are important biomarkers in breast cancer. The performances of FusionQ on cancel line data still showed better specificity and sensitivity in the comparison with another two tools.

**Conclusions:**

FusionQ is a novel tool for fusion detection and quantification based on RNA-Seq data. It has both good specificity and sensitivity performance. FusionQ is free and available at http://www.wakehealth.edu/CTSB/Software/Software.htm.

## Background

Cancer is fundamentally the result of a wide range of genomic alterations. Abnormal chromosome rearrangement is one of its key features and highly involved in hematopoietic diseases. The chromosome breakage and rejoining result in gene fusions, which hybridize the previously separated genes. An increasing number of gene fusions are being considered as important parameters in the diagnosis and prognosis of malignant hematological disorders [1]. A well-known prototype example is the *BCR-ABL1* fusion gene, which has been confirmed as a key pathogenic factor for chronic myeloid leukemia [1]. Recent studies suggest that causal gene fusions may also exist in epithelial-origin carcinomas, and specify the *TMPRSS2-ERG* gene fusion in prostate cancers [2], and *BCAS4-BCAS3* gene fusion in breast cancers [3] as examples. Analysis of these gene fusions from a perspective of genome sequence and structure could provide relevant data that could guide development of improved cancer diagnostics and targeted therapies.

Advances in next-generation sequencing (NGS) technology have imparted a new approach to systematically identify genomic alterations. The sequencing instruments provide a set of deep coverage and base level sequence data, giving a new picture of genes expressed in a cell. Application of one of the NGS approaches, whole transcriptome sequencing (RNA-Seq) technology, allows for the study of functional fusion genes, and gene structures and expressions. Compared to whole-genome sequencing, RNA-Seq focuses on expressed gene fusions, which are very important in cancer development [4]. RNA-Seq technology has been shown to be a powerful tool for gene fusion detection.

Recent gene fusion studies have developed several tools for gene fusion detection. FusionSeq [5] identifies gene fusions from discordant alignments, introducing numerous filters to separate the real fusions from many false-positive ones. However, this method can only detect the fusions between annotated genes, and may miss some fusions due to the incomplete annotation of a complex transcriptome. In addition, it is quite time and space consuming compared to other tools. The deFuse [6] uses dynamic programming to distinguish spanning pairs by split read analysis. However, it operates under the restriction that there are at least five spanning pairs; hence, it is very insensitive to fusions from RNA-Seq datasets with minimal sequence generation. Meanwhile, the classifier to distinguish the true fusions from the false positives is highly related to a fixed training set generated from cell lines. The training set may not be applicable to other cell lines or clinical sample datasets. Fusion-Hunter [7] and TopHat-Fusion [8] use a splice algorithm for gene fusion detection. For splice algorithm, the problem occurs when confirming the alignments of the short sequences around the fusion point [9]. Fusion-Hunter simply aligns the short segments, which will introduce many false positives. TopHat-Fusion searches and merges reads together, but operates under the restriction that there should be 13bp on both sides of fusion points. In addition, all of the aforementioned approaches use the numbers of fusion reads to quantifying the fusion expression levels, which only represent the abundances of small regions around the fusion points.

In this article, we proposed a new tool, FusionQ, which can detect gene fusions, construct the chimerical transcript structures, and estimate their expressions. FusionQ uses a splice algorithm for fusion detection. A new approach, “residual sequence extension”, is proposed in FusionQ to overcome the problem of multiple alignments of short sequences that is commonly seen in splice algorithms. Based on this method, longer sequences can be obtained around the fusion point to confirm their alignments in FusionQ. To reduce the false-positive rate, FusionQ introduces a list of filters, including read number, sequence similarity, read position distribution filter. These filters guarantee FusionQ to obtain results with high specificity. In addition, FusionQ uses a more stable method to quantify fusion expressions. It incorporates the platform of expression estimation tool RSEM (RNA-Seq by Expectation Maximization) [10,11], and uses the Expectation Maximization (EM) algorithm with sparse optimization to estimate chimerical transcript abundance. Furthermore, this abundance quantification can be increase the identification accuracy of FusionQ. Taken together, these features allow FusionQ to provide a more complete view of gene fusion events.

## Methods

In this study, we generated 50 datasets of simulated paired-end RNA-Seq reads using “Eric the simulator” [12]. Each dataset contained 50 simulated gene fusions, with the coverage from 1 to 50. The simulation steps are summarized in the Result Section: comparison on simulated data. Furthermore, we analyzed a published paired-end RNA-Seq dataset (from NCBI Sequence Read Archive [SRA: SRP003186, http://trace.ddbj.nig.ac.jp/DRASearch/study?acc=SRP003186]). It includes data from four breast cancer cell lines (BT474, SKBR3, KPL4, and MCF7), which are known to contain 27 gene fusions. Additionally, we employed a control group from this dataset, which is the RNA-Seq data from normal tissue.

**Figure 1:**
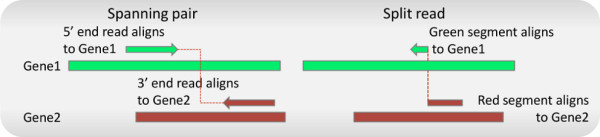
**A spanning pair is 1 paired-end read with discordant alignments.** A split read is a read that contains the fusion point, with the segments separated by the fusion point mapped to different genes. The green and red long rectangles represent the reference sequences of gene1 and gene2, while the green and red arrows represent reads or segments mapped to gene1 and gene2, respectively.

In order to describe FusionQ clearly, some concepts must first be clarified. According to paired-end RNA-Seq technology, a library of cDNA fragments is derived from long RNAs by RNA or DNA segmentation. Short sequences from these paired-ends can be obtained after attaching adaptors to both ends of cDNA fragments. These sequences are termed paired-end reads. Due to the limitation of sequencing length, the middle portion of the cDNA fragments may not be sequenced. These non-sequenced segments are called insert sequences. If a fusion point, which is the joint point of two fused genes, is located in a cDNA fragment, it should exist in either one of the paired-end reads or the insert sequence. A read satisfying the former case is termed a split read, while paired-end reads satisfying the latter case are termed spanning pairs. As to a split read, if the segment on each side of its fusion point can align to a unique gene, the fusion reads are termed as uniquely mapped reads. We graphically describe these two types of fusion reads in Figure 1.

**Figure 2:**
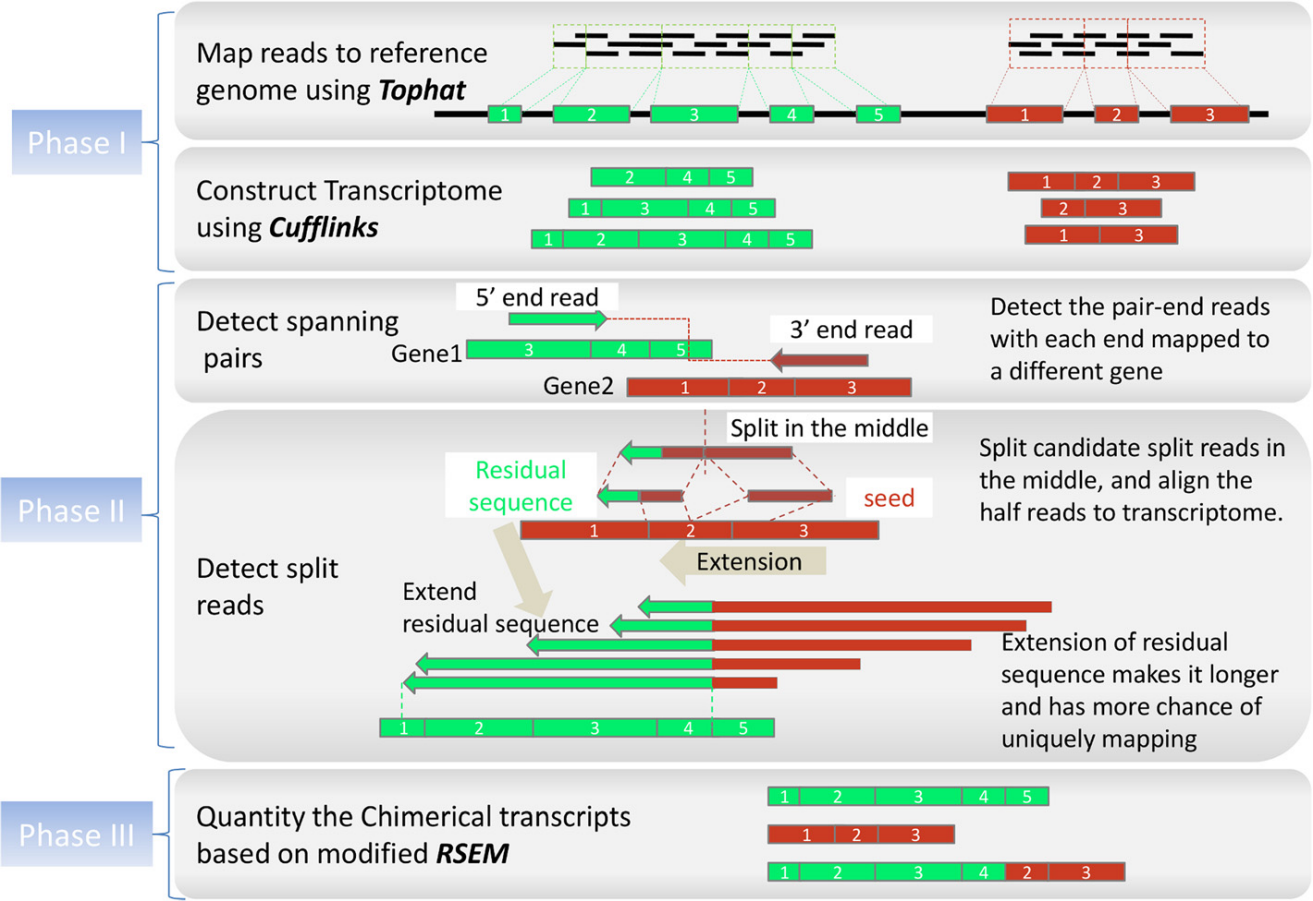
**Flowchart of FusionQ. FusionQ constructs the reference transcriptome directly from the RNA-Seq data, and detects the two types of fusion reads based on it.** The proposed residual sequence extension method makes the short residual sequence longer, and therefore has a higher probability for unique mapping and increases the specificity of the detection results. The chimerical transcript quantification is based on the RSEM algorithm.

The fusion detection process of FusionQ is schematically described in Figure 2.

As seen in Figure 2, FusionQ is composed of three phases:

Phase I Transcriptome construction from RNA-Seq;

Phase II Fusion reads detection based on the constructed transcriptome;

Phase III Chimerical transcript quantification.

The transcript library constructed in phase I contains both known and new transcripts. FusionQ then aligns reads to this constructed transcriptome. As a result, it can detect both known and novel gene fusions. In phase II, FusionQ detects the spanning reads by finding the paired-end reads with discordant alignments, with two ends aligned to different genes, while it detects the split reads by determining if the reads that cannot be aligned to the transcriptome harbor fusion points or not. A residual sequence extension method is proposed in this step, which can largely decrease the false-positive rate in fusion detection. The last phase, quantification of chimerical transcripts, is fulfilled using the Expectation Maximization (EM) algorithm with sparse optimization. A detailed explanation of these phases is as follows:

### Phase I. Transcriptome construction from RNA-Seq

One advantage of RNA-Seq technology is that it can identify transcript structures without knowledge from a pre-existing gene library, and is able to detect novel genes or isoforms. Gene fusion detection from RNA-Seq data should also include the detection of these novel gene fusions to avoid missing fusions due to the incomplete annotation of a complex transcriptome. To address this problem, FusionQ directly constructs the transcriptome from the RNA-Seq data using the transcriptome construction tool, Cufflinks [13]. In addition, a reference annotation to guide the assembly steps is supplied. The output transcriptome by Cufflinks includes all reference transcripts, as well as the novel isoforms. Hence, this approach can report gene fusions between annotated genes and novel genes. Supplying the reference annotation assists in ensuring that low expressed transcripts are not missed and that the constructed transcriptome is more complete. FusionQ then creates a Bowtie index of the transcriptome using the Bowtie-build program [14].

### Phase II. Fusion reads detection based on the constructed transcriptome

1. Mapping

Based on the created index from phase I, FusionQ first uses Bowtie to map all reads to the constructed transcriptome. The paired-end reads with concordant alignments and reasonable insertion lengths are filtered out from the consideration of fusion reads. The paired-end reads with discordant alignments are considered as spanning reads. Some paired-end reads having neither ends aligned to the transcriptome are split into halves. If each end of one paired-end read has half mapped to the transcriptome, and the alignments are discordant, these paired-end reads are still considered as spanning reads. If one paired-end read has one end mapped to the transcriptome while the other not, the non-mapped end may harbour a fusion point. This type of non-mapped read is selected as a candidate split read. This mapping step allows a number of mismatches according to the transcriptome, considering sequencing errors and single nucleotide polymorphisms.

2. Splitting reads into halves

The candidate split reads are split in the middle. For the purposes of this study, it was assumed that a split read harbors only one fusion point. If this read is then split in the middle, at least half can align to the transcriptome. This half read is called a seed. To guarantee that most half reads do not have too many mapping positions, read lengths are restricted to be more than 50bp, because 25bp reads have a high probability to align to unique genes of the constructed transcriptome. This conclusion is based on the following example. Ten thousand (10000) 25bp reads that can align to a constructed transcriptome were chosen at random. Almost 75% of them could align uniquely, with only less than 5% of the reads mapped to more than three genes. Therefore, the detected seeds are restricted so as to have no more than one mismatch based on the transcriptome.

3. Fusion breakpoint detection

In the following extension step, the detected seeds are extended, nucleotide by nucleotide. Extension is stopped when further extension results in no mapping position. This stop point is regarded as the fusion breakpoint. The remaining segment that does not undergo seed extension is called a residual sequence. The implying genes of the seed and residual sequence are the gene fusion partners. Clearly, the length of the residual sequences is less than half of the read length. Consequently, it cannot be guaranteed that most of them align to less than three genes. Some split reads may be missed by simply disregarding some of the short residual sequences. However, if those residual sequences have too many mapping positions, the false-positive rate may increase. Consequently, while considering both detection sensitivity and specificity, the problem of how best to determine implying genes from the residual sequences presents. Residual sequence extension is therefore proposed to address this concern.

4. Residual sequence extension

**Figure 3:**
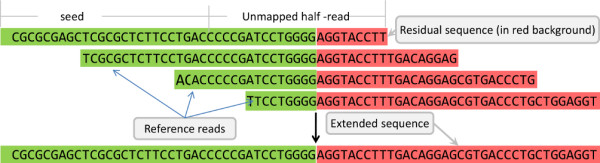
Residual sequence extension step.

Based on deep sequencing, a split read should find some overlap reads harboring the same fusion point. If a fusion is only expressed in a single read, it cannot be determined if the detected fusion is actual or an internal error of the software. Therefore, as to a split read, the 20bp segment with the fusion point in the middle is regarded as a contig. If the residual sequence is less than 10bp, the 20bp segment containing this residual sequence is regarded as the contig. All of the non-mapped reads other than the candidate split reads are then searched, and all reads that have this contig are selected. A set of these selected reads may have concordant overlap with this split read. They would be termed reference reads to the split read. The reference reads are then merged together to several longer sequences. The implying gene of the short residual sequence is decided by these longer sequences. This step is graphically described in Figure 3.

5. Filtering false positives

a) Reads number filter. After detecting the spanning pairs and split reads, the results are combined, and the reads that are less possible to be true fusion reads are filtered out. In the two-partner fusion transcripts reported from a split read, one should also be the aligned transcript of its mate read. The implying fusion points of the spanning pairs and split reads should be concordant. In addition, the fusions are discarded if either their supporting spanning pairs, split reads or fusion numbers are lower than the minimum numbers user specified.

b) Similarity filter. Some detected fusion junctions may be reported between two paralogous genes. They often result from misalignment due to their homology. This homology could be quantitatively measured by the degree of sequence match between the two genes. The matching degree is called *similarity*. The Water output from the EMBOSS [15] pipeline is used to calculate similarity between every pair of reported partner isoforms. The fusion is discarded if similarity is more than 50%.

c) Distribution filter. This filter is designed based on the hypothesis that the distributions of the positions of the reads involved in one transcript are similar. Firstly, a large number of “real” transcripts are randomly selected, which have reads of both ends mapped to transcriptome. The positions of the supporting reads for these transcripts are collected and merged as “background positions”. Secondly, the distributions similarity is estimated between the positions of the supporting reads for each of these transcript and the “background positions” using Kolmogorov–Smirnov (K-S) test. A list of p-values is then obtained, and their distribution is defined as the distribution of p-values (density function of p-values). The null hypothesis is that the p-value of K-S test between the positions of supporting reads for a real transcript and “background positions” obeys the distribution of p-values. In consequence, as to each detected fusion, the positions of the spanning pairs are recorded, and the p-value of K-S test between each end read positions and “background positions” is calculated. Because the two end reads are corresponding to two fused transcripts, according to the null hypothesis, both the two p-values should obey the distribution of p-values. If either p-value falls to the significantly low level in the p-value distribution (significant level is 0.025), the corresponding fusion is discarded.

In order to reduce false positive rate of fusion detection, a list of filters are performed on the detected fusions in this step.

### Phase III. Chimerical transcripts quantification

In addition to the filters above, the identification accuracy of FusionQ is further improved by quantifying the abundance of chimerical transcripts. Generally speaking, highly expressed gene fusions are more important than those under a low expression levels. Most tools use the number of reads supporting the fusion junction to determine the expression of this fusion. However, this number only reflects the expression level of the short region around the fusion point. Some chimerical transcripts with higher expression levels may have many reads, but the reads do not contain the fusion points, resulting in low supporting read counts. In addition, due to the complexity of the transcriptome, some reads may be mapped to several genes. Simply treating them as the supporting reads of certain fusion genes may cause misalignment and mistakenly increase their supporting reads counts. In order to provide more reliable expression levels for gene fusions, the quantifications should be based on the whole chimerical transcript. Here, the framework of RSEM (RNA-Seq by Expectation Maximization) for chimerical transcripts quantification can be used by adding a L1 norm constraint to the number of expressed fusion transcripts.

The chimerical transcript set is denoted as $\Theta ={\left\{\theta_{i}\right\}}_{i=1}^{M}$. All of the paired-end reads are denoted as X**.** The goal is to determine the abundances of $\overset{⌢}{\Theta }$ that can maximize the sampling likelihood of X. For one transcript *i*_,_ it has a subset of supporting reads $\prod_{x_{n}}^{i}$ aligned to it. The paired-end reads $x_{n}\left(x_{n}\in \Pi_{x_{n}}^{i}\right)$ may come from multiple fragments $\Pi_{x_{n}}$. The probability of the *x*_*n*_ takes the *j*^*th*^ fragment of $\Pi_{x_{n}}$ is $P\left(\Pi_{x_{n}}^{j}\right)$. For each paired-end read *x*_*n*_, the probability of one fragment coming from *i* is *θ*_i_. *θ*_i_ is normalized by $P\left(\Pi_{x_{n}}^{j}\right)$ as well as the length of transcript *l*_*i*_. As to a paired-end read *x*_*n*_, the two ends are $x_{n}^{\alpha }$ and $x_{n}^{\beta }$, with the insert sequence denoted as $x_{n}^{\gamma }$. *P*(*x*_*n*_|*Z*_*nij*_ = 1) is the probability to get observation *x*_*n*_ from this fragment, where the hidden indicator random variable *Z*_*nij*_ = 1 means the alignment of *j*^*th*^ read $\Pi_{x_{n}}$ aligns to transcript *i*. Then, taking all reads and all possible fragments of each read together, we aim to find *Ψ* that maximizes the likelihood of the sampling of the paired-end reads in X. This likelihood is

$$P\left(X|\Theta \right)=\prod_{n=1}^{N}\sum_{i=1}^{M}\sum_{\pi_{x_{n}}^{j}\in \prod_{x_{n}}^{i}}\frac{\theta_{i}}{l_{i}}P\left(\pi_{x_{n}}^{j}\right)P\left(x_{n}|Z_{\mathrm{nij}}=1\right)$$

Where

$$P\left(x_{n}|Z_{\mathrm{nij}}=1\right)=P\left(x_{n}^{\alpha }|Z_{\mathrm{nij}}=1\right)\cdot P\left(x_{n}^{\beta }|Z_{\mathrm{nij}}=1\right)\cdot P_{d}\left(x_{n}^{\gamma }\right)\cdot P\left(\psi \right)$$

$P_{d}\left(x_{n}^{\gamma }\right)$ follows the empirical normal distribution of the mate-pair distance. The probability of the sequenced end reads taking the *j*-th element of $\prod_{x_{n}}$, $P\left(x_{n}^{\alpha }|Z_{\text{nij}}=1\right)$ and $P\left(x_{n}^{\beta }|Z_{\mathrm{nij}}=1\right)$, should be related with their sequence similarities to the reference genome and their base call quality score [16]. *P*(*ψ*) is the probability of the junction crossed by one alignment of paired-end reads which can be calculated from the observed reads.

The Expectation-Maximization (EM) algorithm is then applied to maximize the log likelihood of *P*(**X**|*Θ*). The key of the EM algorithm is the expected value of the complete data log likelihood function, given current values for the parameters. The complete data log likelihood with group sparsity constraints is written as $\text{log}P\left(X,Z|\Theta \right)=\sum_{n,i}\sum_{\pi_{x_{n}}^{j}\in \prod_{x_{n}}^{i}}\frac{\theta_{i}}{l_{i}}\text{log}\left[\frac{\theta_{i}}{l_{i}}P\left(\pi_{x_{n}}^{j}\right)P\left(x_{n}|Z_{\mathrm{nij}}=1\right)\right]-\sum_{k=1}^{K}\alpha_{k}||\Phi_{k}|{|}_{1}$ where *Φ*_*k*_ are the abundance of transcripts with detected junctions or fusions.

The *Q* function of the EM algorithm is $Q\left(\Theta |\Theta^{\left(t\right)}\right)=E_{Z|x,\Theta^{\left(t\right)}}\left[\text{log}P\left(x,z|\Theta \right)\right]=\sum_{n,i,j}E_{Z|x,\Theta^{\left(t\right)}}\left[Z_{\mathrm{nij}}\right]\text{log}\left(\frac{\theta_{i}}{l_{i}}P\left(x_{n}|Z_{\mathrm{nij}}=1\right)\right)-\lambda \sum_{k\in F}\text{log}|\theta_{k}|$.

During E-step, we calculate the expected values of the *Z*_*nij*_ variables,

$$\begin{array}{ll} E_{Z|x,\Theta^{\left(t\right)}}\left[Z_{\mathrm{nij}}\right] & =P\left(Z_{\mathrm{nij}}=1|x,\Theta^{\left(t\right)}\right)\\ & =\frac{\left(\theta_{i}^{\left(t\right)}/l_{i}\right)P\left(x_{n}|Z_{\mathrm{nij}}=1\right)}{\sum_{i^{'},j^{'}}\left(\theta_{i^{'}}^{\left(t\right)}/l_{i^{'}}\right)P\left(x_{n}|Z_{ni^{'}j^{'}}=1\right)} \end{array}$$

In the M-step, we maximize the likelihood of *Q* and estimate $\overset{⌢}{\Theta }$ by the Lagrange multiplier method.

Based on this method, reads are mapped to the optimized positions. The corresponding expression levels of the detected fusions are then more reliable. Some of these fusions may have expression levels approximate to zero. That would indicate that their supporting reads are the results of misalignment. These fusions are likely to be artificial and could be disregarded. In this way, the fusion identification accuracy is further improved.

## Results

In this section, the performance of FusionQ was tested on simulated data and cancer cell line data. Two fusion detection tools, “deFuse”[6] and “TopHat-Fusion” [8]were adopted for comparison.

### Comparison on simulated datasets

Fifty simulated paired-end RNA-Seq datasets were generated using “Eric the Simulator” [12]. Each dataset was composed of two millions “background” reads and a set of “broken exons” (BE) fusion reads. The “background” reads were the randomly selected ones from a published RNA-Seq dataset of the untreated human pulmonary microvascular endothelial cells [17]. The average length of the original cDNA fragments was ~164bp and standard deviation was ~48. No fusion should exist in these background reads. The BE fusion reads were generated from the 5’- and 3’- end of 50 simulated BE chimerical transcripts using wgsim (http://github.com/lh3/wgsim) (with –d 164 –r 0.0001 –R −0.001 –s 48). Broken exon (BE) means that the fusion breakpoints are randomly chosen without knowledge of known splicing sites of fused genes, in which case, the breakpoints may exist on exons. The coverage levels of the 50 simulated chimerical transcripts were from ranged 1 to 50. These fused transcript partners were the randomly selected ones from Ensemble Transcriptome database version 65. The read length was 50bp.

Next, the three tools, FusionQ, deFuse, and TopHat-Fusion, were performed on these 50 simulated datasets using the same computational sources provided by the Stampede server of “Texas advanced computing center”. The number of threads was set as 16 as to all these three tools. We compared detection sensitivity and specificity of the three tools. Sensitivity is defined as the number of true fusions detected divided by total number of true fusions (50), while specificity is defined as the number of true fusions detected divided by number of all fusions detected. The statistics were shown in Table 1.

**Table 1:** Performance comparison among FusionQ, deFuse and TopHat-Fusion based on simulated data sets

**Method**	**Total fusion No.**	**True fusion No.**	**Specificity**	**Sensitivity**	**Time/h**
FusionQ	63	40	63.5%	80%	4.5
deFuse	62	37	60%	74%	3.4
TopHat-Fusion	32	26	81%	52%	0.67

Specificity: Number of true fusions detected divided by number of all detect fusions.

Sensitivity: Number of true fusions detected divided by total number of true fusions (50).

Besides, ROC curves were drawn based on 50-quantiles. Firstly, the results of FusionQ, deFuse, and TopHat-Fusion were sorted in ascending order by fusion expression levels, estimated probabilities, and number of fusion reads, respectively. Then, the ROC curves were plotted with each point representing a pair of true positive rate (TPR) and false positive rate (FPR) when only the last kth 50-quantile results were considered. TPR and FPR were defined as follows: as to the last kth 50-quantile, $\text{TPR}=\frac{\mathrm{TP}}{\mathrm{TP}+\mathrm{FN}}$ and $\text{FPR}=\frac{\mathrm{FP}}{\mathrm{FP}+\mathrm{TN}}$**,**

where TP and FP were the number of true and false fusions in the last kth 50-quantile results, and TN and FN were the number of true and false fusions in the rest of the results.

**Figure 4:**
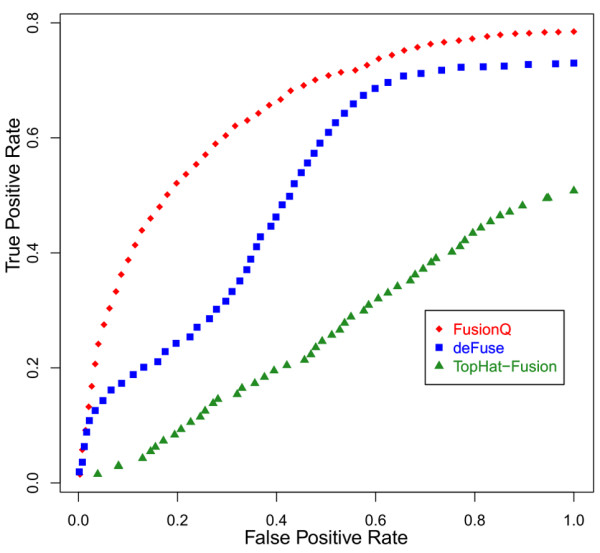
ROC curves obtained from the results of FusionQ, deFuse and TopHat-Fusion.

According to Table 1 and Figure 4, TopHat-Fusion cost the least time. However, it had the lowest detection sensitivity, with only 52% true fusions detected. Although FusionQ cost more time for computation, it outperformed the other two programs, and excelled deFuse in both sensitivity and specificity. The time-consuming step of FusionQ is the transcript expression estimation. However, it is a unique function that FusionQ has but the other two tools do not. It can estimate the fusion transcript expression levels and largely reduces the false positive rate. As to these 50 simulated data sets, after the first three filters described in Method Section was performed, around 65% of the rest false fusions were excluded by means of expression filters. True fusions were seldom filtered out.

**Figure 5:**
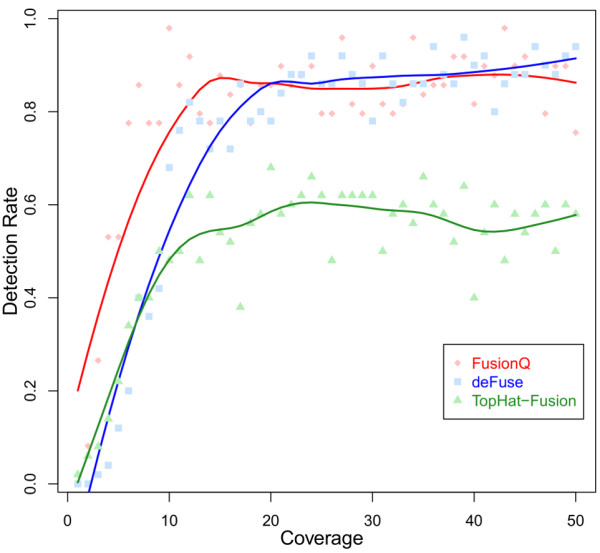
The detection ability to different coverage levels of FusionQ, deFuse, and TopHat-Fusion.

In addition, we estimated how the read coverage affected the detection ability. Among the 50 simulated data sets, each contained fusions with coverage from 1 to 50. As to the fusion transcript of each coverage level, we counted the number of times that the tools could detect it among the 50 data sets. Hence, we obtained the detection rates of the three tools as to each coverage transcript. The relations between the detection rates to chimerical transcript coverage of the three tools were shown in Figure 5.

As shown in Figure 5, FusionQ and deFuse showed greater detection ability than Tophat-Fusion. FusionQ was better than deFuse when read coverage was low.

### Comparison on cancer cell line datasets

In the following, FusionQ was tested on published paired-end RNA-Seq data from four breast cancer cell lines (BT474, SKBR3, KPL4, and MCF7). These cell lines are known to contain 27 gene fusions [18]. Data could be obtained from the NCBI Sequence Read Archive [SRA: SRP003186, http://trace.ddbj.nig.ac.jp/DRASearch/study?acc=SRP003186]. The control group of this dataset was also employed. It was the RNA-Seq data from normal tissue, which should have no gene fusions. A parallel computation was incorporated into the program to increase detection speed. As the dataset may contain many duplicate reads resulting from the PCR, quality control on reads level was performed to remove them. This filtered out the unnecessary reads and reduced the computational time. This method may reduce the reads coverage level, which could test our program on low coverage level conditions. Table 2 lists the description of these datasets both before and after quality control.

**Table 2:** Paired-end RNA-Seq data in the test dataset

**Cancer type**	**Sample names**	**Fragment lengths**	**Read length**	**Read numbers before QC**	**Read numbers after QC**
Breast cancer	BT474	100, 200	50	21,423,697	9,416,283
	SKBR3	100, 200	50	18,140,246	7,190,185
	KPL4	100	50	6,796,443	2,616,395
	MCF7	100	50	8,409,785	4,223,773
Normal tissue	Normal	100	50	4,564,298	2,701,076

When running FusionQ, both the fusion partners were restricted to having at least 5bp involved in one split read. In addition, the detected split reads could have10bp segments around the fusion points having no mapping positions in the transcriptome. All of the reported fusions should have spanning pairs and split reads. Furthermore, the mismatches between the reads and transcriptome were restricted to less than three, while those between the seeds and transcriptome to less than one. Because quality control was performed on the data from the breast cancer cell lines and normal tissues, the detected fusion numbers should be smaller. As a result, fusions that have more than one split read and one spanning pair were reported, but the total numbers of supporting reads are more than three. Additionally, UCSC hg19 was used as the reference human genome [19], and the reference transcriptome was constructed using “TopHat” [20], then Cufflinks (v 1.3.0). When using Cufflinks, a reference annotation for UCSC hg19 (.gtf) was supplied to guide the assembly steps.

Besides FusionQ, two fusion detection tools (deFuse” and “TopHat-Fusion) were applied to datasets after performing quality controls, and their performances were compared with FusionQ. The results of TopHat-Fusion were restricted to containing more than one split read, one spanning pair, and more than three supporting fusion reads, which is the same as FusionQ. deFuse requires at least five spanning pairs to nominate a gene fusion to the classifier. In consequence, deFuse was restricted to having at least one split read and five spanning pairs. Other parameters of these two tools are by default. After computation, the detected fusions by these three tools were listed in Additional files 1: Table S1, Additional file 2: Table S2, Additional file 3: Table S3. Table 3 provides a summary of the detection results.

**Table 3:** Summary of the detected fusions by the three tools

	**FusionQ**	**deFuse**	**TopHat-Fusion**
Total number of fusions	298	1932	111
No. of detected fusions that are reported/ No. of documented fusions	81%(22/27)	74%(20/27)	59%(16/27)

Comparison of the fusion number by FusionQ, deFuse, and TopHat-Fusion. Empty cells denote a failure of the tool to report a particular fusion.

In total, TopHat-Fusion reported 111 fusions and FusionQ reported 298 fusions, while deFuse reported 1932 fusions, as shown in Table 3. This demonstrated that FusionQ reported less false-positive fusions than those reported by deFuse. Among the 27 documented fusions, FusionQ detected 22 of them, while deFuse and TopHat-Fusion detected 20 and 16 fusions, respectively. This is quite different from the results on data before quality control. While the quality control will significantly reduce the number of false-positives, its performing will result in a corresponding decrease in reads coverage, TopHat-Fusion showed unstable performance, and could not predict some very low coverage reads. FusionQ showed more sensitivity in this occasion. The detection results of the 27 reported fusions by the three tools are presented in Table 4.

**Table 4:** Fusion detection performance comparison based on cancer cell line data sets

**Library Name**	**Fusion genes**	**deFuse**		**TopHat-Fusion**		**FusionQ**	
		**split reads**	**spanning reads**	**split reads**	**spanning reads**	**split reads**	**spanning reads**
SKBR3	TATDN1-GSDMB	67	51	94	27	144	19
SKBR3	RARA-PKIA	7	12	6	7	4	11
SKBR3	ANKHD1-PCDH1	7	14	4	7	6	8
SKBR3	CCDC85C-SETD3	4	6			3	5
SKBR3	SUMF1-LRRFIP2	9	16			3	9
SKBR3	CSE1L-ENSG00000236127	7	19				
SKBR3	WDR67-ZNF704					2	1
SKBR3	CYTH1-EIF3H	5	27			7	22
SKBR3	DHX35-ITCH	4	5			4	1
SKBR3	NFS1-PREX1						
BT474	ACACA-STAC2	20	20	14	44	8	27
BT474	RPS6KB1-SNF8	28	31	24	32	9	11
BT474	VAPB-IKZF3	13	31	26	27	8	17
BT474	ZMYND8-CEP250	7	16	10	11	8	17
BT474	RAB22A-MYO9B	9	5	10	1	3	4
BT474	SKA2-MYO19	5	6	10	5	3	3
BT474	DIDO1-KIAA0406			1	3		
BT474	STARD3-DOK5			3	3	1	3
BT474	LAMP1-MCF2L						
BT474	GLB1-CMTM7			2	6	1	3
BT474	CPNE1-PI3						
MCF7	BCAS4-BCAS3	67	77	54	39	14	40
MCF7	ARFGEF2-SULF2	25	24	5	8	13	11
MCF7	RPS6KB1-TMEM49	6	8	3	3	2	1
KPL4	BSG-NFIX	23	18	4	11	3	13
KPL4	PPP1R12A-SEPT10	6	8			5	2
KPL4	NOTCH1-NUP214	2	6			3	3

As shown in Table 4, FusionQ includes gene fusion *WDR67-ZNF704*, which is missed by the other two methods. This demonstrates that FusionQ has the highest sensitivity. The number of reads detected by FusionQ and TopHat-Fusion are comparable, although FusionQ had much higher sensitivity than TopHat-Fusion. deFuse seems to detect a higher number of fusion reads than that done by FusionQ and TopHat-Fusion. This is probably because FusionQ and TopHat-Fusion use Bowtie mapping, of which the mapping criteria is more strict, whereas deFuse uses dynamic programming to decide the sequence alignment, of which the criteria is loose, and false fusions may not be completely ruled out. In summary, FusionQ could report more fusions among all reported when the reads coverage is low. This demonstrated the advantage of FusionQ in detection sensitivity. Meanwhile, the total number of fusions detected by FusionQ is much less than that of deFuse, which means that FusionQ could report fusions with less false positives than deFuse.

### Fusion structure construction

**Figure 6:**
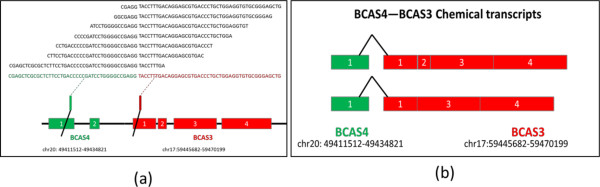
**Structure of *BCS4-BCAS3*. The left figure shows the construction process.** The reads in black are the original reads. The bars of each read are the fusion points. Using our algorithm, the reads are merged together, with the green one involved in *BCAS4*, and the red one involved in *BCAS3*. The fusion has two different chemical transcripts, which are shown in the right figure.

Here, the *BCAS4-BCAS5* gene fusion in the MCF7 cell line is used as an example to demonstrate fusion structure construction. This fusion results from the first exon of *BCAS4* fused to exon 1 of *BCAS3*, as shown in Figure 6(a). Only a few fusion reads used in this example. The fusion points are the bars of each read, separating each read into two segments. Using the “residual sequence extension” algorithm, the two segments of each read can be extended into longer ones. The extended segments are uniquely mapped to *BCAS4* and *BCAS3*, respectively. Moreover, Figure 6(b) describes all of the possible chemical transcripts of *BCAS4-BCAS3.*

### Abundance quantification by FusionQ

The proposed scoring function in Phase II is based on the numbers and distributions of supporting reads. Fusions with higher scores have better features around the fusion point, and are more likely to be true fusions. The scoring function judges the fusions from a local view. Furthermore, to profile the fusions from a global view, the abundance of the detected fusions is quantified using the method described in Phase III. This method could determine the optimized mapping positions of each read, as well as the average expression level of all transcripts, including the chimerical transcripts. Fusions with very low expression levels could be disregarded after the analysis. This step improves the accuracy of the fusion detection.

Here, the detection results based on the data from the KPL4 cell line are presented. In total, 15 fusion genes were detected, including 22 fusion transcripts. The ranking score and expression level of every detected fusion are shown in Table 5.

**Table 5:** Expression estimation of fusions from KPL4 cell line

**Fusion Names**	**Split reads**	**Spanning Pairs**	**expression**
**NFIX-BSG**	**3**	**13**	**304.05**
ACIN1-C14ORF119	2	13	163.28
BCL9L-CUFF.3497	8	4	530.01
BSG-NFIC	9	5	13.08
**NUP214-NOTCH1**	**3**	**3**	**157.70**
BRWD1-LOC100132288	5	3	93.92
**SEPT10-PP1R12A**	**5**	**2**	**145.35**
WNT5A-CUFF.12313	5	2	243.37
LRRIQ1-SEC14L1	1	5	0
SGK269-KIAA1328	3	3	18.38
ASTN2-CUFF.8591	1	4	0
C9ORF129-CUFF.14016	3	2	33.26
UTS2D-PTGR1	3	2	2.58

Gene name with the prefix “CUFF” are novel genes predicted by Cufflinks.

The reported gene fusions are in bold.

As shown in Table 5, all of the three reported fusions in bold have higher expression levels, although one of them, *NUP214-NOTCH1*, does not have many supporting reads. This confirms that the expression estimation by the EM algorithm is significantly more reliable than read counts. In addition, some fusions with more supporting reads could have relatively low expression, such as *BSG-NFIC*. The supporting reads of these fusions may contain some misalignments. Furthermore, the misalignment could cause artificial fusion junctions. However, after abundance quantification, the expression levels of these artificial fusions are approximate zeros. As a result, this quantification could further improve the accuracy of fusion identification.

Among the results listed in Table 5, the three reported fusions along with four other fusions (*ACIN1-C14ORF, BCL9L-CUFF.3497, BRWD1-LOC100132288, and WNT5A-CUFF.12313*) have higher expression levels. These fusions are mostly likely to be true fusions based on the FusionQ program. Besides, the gene partners with prefix “CUFF” are novel genes predicted by Cufflinks. The three fusions, *BSG-NFIC, SGK269-KIAA1328*, and *C9ORF129-CUFF.14016*, have relatively lower expressions. Their scores are relatively low. These fusion genes are judged as true fusions by FusionQ. As to the three fusions, *LRRIQ1-SEC14L1*, *ASTN2-CUFF.8591,* and *ASTN2-CUFF.8591*, the expression levels are approximate zeros. This means that these fusion junctions are artificial, and could be disregarded. In summary, the scores and expression values could help to select the potential fusions for further validation.

## Discussion and Conclusion

In this study, we developed a novel tool, *FusionQ,* to detect gene fusions, construct the chimerical transcripts structures, and estimate their expressions. FusionQ uses a splice algorithm for fusion detection. To determine the position of short segments in the transcriptome, we proposed a new approach using a residual sequence extension algorithm. The short segments of the reads are extended by aggregating their overlapping reads. This approach makes the prediction more precise. We also incorporated filters to the detected results, which reduced the false-positive rate. Moreover, instead of describing a fusion expression level using the number of supporting reads, we used sparse optimization to quantify the abundance of a fusion transcript. Results show that this method is more reliable than using the supporting read count. The abundance quantification also further improves the identification accuracy of our approach. We compared FusionQ with two current fusion detection tools, deFuse and TopHat-Fusion using simulated data and cancer cell line data. FusionQ showed better detection capacity than TopHat-Fusion in low coverage situations. Its performance was comparable with deFuse, but the total number of fusions reported by FusionQ is much lower than that by deFuse, as shown in Table 3. Hence, FusionQ could report fusions with a lower false-positive rate. As abnormal chromosome translocation is one of the important pathogenic factors in cancer development, our FusionQ approach will facilitate accurate detection of gene fusions for disease diagnosis and identification of potential targets for gene therapy.

### Availability and requirements

**Project name**: FusionQ: a novel approach for detection and quantification of gene fusions from paired-end RNA-Seq

**Project home page**: http://www.wakehealth.edu/CTSB/Software/Software.htm

**Operating system:** 64-bit Linux (The program has been tested on Ubuntu, Debian, and Centos)

**Programming language**: C++, Perl and R

**Other requirements**: Boost C++ libraries,1.42.0 and above.

## Competing interests

The authors declare that they have no competing interests.

## Authors' contributions

CL participated to study conception, tool development, and drafted the manuscript. XZ participated to study conception and improved the manuscript. CC participated to study conception, and improved the manuscript. JM helped to study conception. All authors read and approved the final manuscript.

## Supplementary Material

Additional file 1: Table S1 Fusion Reports from FusionQ. Click here for file

Additional file 2: Table S2 Fusion Reports from Tophat-Fusion. Click here for file

Additional file 3: Table S3 Fusion Reports from deFuse. Click here for file
