# Supplementary material for: FusionQ: a novel approach for gene fusion detection and quantification from paired-end RNA-Seq
Source: BMC Bioinformatics. 2013 Jun 15;14:193. doi: 10.1186/1471-2105-14-193 (PMC3691734; doi:10.1186/1471-2105-14-193)
Supplement: Additional file 2: Table S2 — Fusion Reports from Tophat-Fusion. [file 1471-2105-14-193-S2.doc]

**Supplement Table 2: Fusion Report From TopH**at-Fusion

| Sample_Name | Gene_1 | Chr_1 | Position_1 | Gene_2 | Chr_2 | Position_2 | Span_Reads | Mate_Pairs | Split_Reads |
| --- | --- | --- | --- | --- | --- | --- | --- | --- | --- |
| SK-100 | TATDN1 | chr8 | 125551165 | GSDMB | chr17 | 38062234 | [2](../../../../J:%5CTophatFusion_for_Breast_Cancer%5C1span%5CSKBR3_100.html#read_3) | [13](../../../../J:%5CTophatFusion_for_Breast_Cancer%5C1span%5CSKBR3_100.html#pair_3) | 4 |
| SK-100 | TATDN1 | chr8 | 125551167 | GSDMB | chr17 | 38066175 | [4](../../../../J:%5CTophatFusion_for_Breast_Cancer%5C1span%5CSKBR3_100.html#read_6) | [13](../../../../J:%5CTophatFusion_for_Breast_Cancer%5C1span%5CSKBR3_100.html#pair_6) | 8 |
| SK-100 | TATDN1 | chr8 | 125551262 | GSDMB | chr17 | 38062234 | [7](../../../../J:%5CTophatFusion_for_Breast_Cancer%5C1span%5CSKBR3_100.html#read_4) | [13](../../../../J:%5CTophatFusion_for_Breast_Cancer%5C1span%5CSKBR3_100.html#pair_4) | 15 |
| SK-100 | TATDN1 | chr8 | 125551264 | GSDMB | chr17 | 38062522 | [5](../../../../J:%5CTophatFusion_for_Breast_Cancer%5C1span%5CSKBR3_100.html#read_5) | [13](../../../../J:%5CTophatFusion_for_Breast_Cancer%5C1span%5CSKBR3_100.html#pair_5) | 7 |
| SK-100 | TATDN1 | chr8 | 125551264 | GSDMB | chr17 | 38066175 | [19](../../../../J:%5CTophatFusion_for_Breast_Cancer%5C1span%5CSKBR3_100.html#read_7) | [13](../../../../J:%5CTophatFusion_for_Breast_Cancer%5C1span%5CSKBR3_100.html#pair_7) | 31 |
| SK-100 | PCBP2 | chr12 | 53858636 | FLJ39080 | chr8 | 75515898 | [2](../../../../J:%5CTophatFusion_for_Breast_Cancer%5C1span%5CSKBR3_100.html#read_12) | [75](../../../../J:%5CTophatFusion_for_Breast_Cancer%5C1span%5CSKBR3_100.html#pair_12) | 3 |
| SK-100 | ENSG00000248530 | chr3 | 131245709 | BCL2L12 | chr19 | 50172276 | [2](../../../../J:%5CTophatFusion_for_Breast_Cancer%5C1span%5CSKBR3_100.html#read_16) | [6](../../../../J:%5CTophatFusion_for_Breast_Cancer%5C1span%5CSKBR3_100.html#pair_16) | 3 |
| SK-100 | CAP1 | chr1 | 40533306 | ENSG00000232004 | chr10 | 44100913 | [1](../../../../J:%5CTophatFusion_for_Breast_Cancer%5C1span%5CSKBR3_100.html#read_10) | [46](../../../../J:%5CTophatFusion_for_Breast_Cancer%5C1span%5CSKBR3_100.html#pair_10) | 1 |
| SK-100 | ANKHD1 | chr5 | 139825559 | PCDH1 | chr5 | 141234000 | [2](../../../../J:%5CTophatFusion_for_Breast_Cancer%5C1span%5CSKBR3_100.html#read_17) | [3](../../../../J:%5CTophatFusion_for_Breast_Cancer%5C1span%5CSKBR3_100.html#pair_17) | 3 |
| SK-100 | CYTH1 | chr17 | 76778283 | EIF3H | chr8 | 117768257 | [2](../../../../J:%5CTophatFusion_for_Breast_Cancer%5C1span%5CSKBR3_100.html#read_14) | [4](../../../../J:%5CTophatFusion_for_Breast_Cancer%5C1span%5CSKBR3_100.html#pair_14) | 2 |
| SK-100 | PRDX5 | chr11 | 64087339 | ENSG00000242364 | chr3 | 73697738 | [5](../../../../J:%5CTophatFusion_for_Breast_Cancer%5C1span%5CSKBR3_100.html#read_13) | [16](../../../../J:%5CTophatFusion_for_Breast_Cancer%5C1span%5CSKBR3_100.html#pair_13) | 4 |
| SK-100 | RNF6 | chr13 | 26795652 | FOXO1 | chr13 | 41192771 | [1](../../../../J:%5CTophatFusion_for_Breast_Cancer%5C1span%5CSKBR3_100.html#read_0) | [4](../../../../J:%5CTophatFusion_for_Breast_Cancer%5C1span%5CSKBR3_100.html#pair_0) | 0 |
| SK-100 | RNF6 | chr13 | 26795971 | FOXO1 | chr13 | 41192773 | [2](../../../../J:%5CTophatFusion_for_Breast_Cancer%5C1span%5CSKBR3_100.html#read_1) | [4](../../../../J:%5CTophatFusion_for_Breast_Cancer%5C1span%5CSKBR3_100.html#pair_1) | 0 |
| SK-100 | BAT1 | chr6 | 31499072 | ENSG00000254406 | chr11 | 119692419 | [2](../../../../J:%5CTophatFusion_for_Breast_Cancer%5C1span%5CSKBR3_100.html#read_15) | [10](../../../../J:%5CTophatFusion_for_Breast_Cancer%5C1span%5CSKBR3_100.html#pair_15) | 1 |
| SK-100 | RARA | chr17 | 38465534 | PKIA | chr8 | 79485042 | [5](../../../../J:%5CTophatFusion_for_Breast_Cancer%5C1span%5CSKBR3_100.html#read_8) | [2](../../../../J:%5CTophatFusion_for_Breast_Cancer%5C1span%5CSKBR3_100.html#pair_8) | 5 |
| SK-100 | ACTN4 | chr19 | 39207954 | ACTN1 | chr14 | 69357003 | [3](../../../../J:%5CTophatFusion_for_Breast_Cancer%5C1span%5CSKBR3_100.html#read_9) | [1](../../../../J:%5CTophatFusion_for_Breast_Cancer%5C1span%5CSKBR3_100.html#pair_9) | 5 |
| SK-100 | NASP | chr1 | 46070687 | ENSG00000254777 | chr8 | 61852322 | [3](../../../../J:%5CTophatFusion_for_Breast_Cancer%5C1span%5CSKBR3_100.html#read_11) | [15](../../../../J:%5CTophatFusion_for_Breast_Cancer%5C1span%5CSKBR3_100.html#pair_11) | 4 |
| SK-100 | ATR | chr3 | 142170161 | EIF2AK1 | chr7 | 6077055 | [1](../../../../J:%5CTophatFusion_for_Breast_Cancer%5C1span%5CSKBR3_100.html#read_18) | [9](../../../../J:%5CTophatFusion_for_Breast_Cancer%5C1span%5CSKBR3_100.html#pair_18) | 1 |
| SK-100 | XBP1 | chr22 | 29192181 | REEP5 | chr5 | 112221156 | [7](../../../../J:%5CTophatFusion_for_Breast_Cancer%5C1span%5CSKBR3_100.html#read_2) | [1](../../../../J:%5CTophatFusion_for_Breast_Cancer%5C1span%5CSKBR3_100.html#pair_2) | 2 |
| [SK-200](../../../../J:%5CTophatFusion_for_Breast_Cancer%5C1span%5CSKBR3_.html#fusion_11) | ANKHD1 | chr5 | 139825557 | PCDH1 | chr5 | 141234002 | [2](../../../../J:%5CTophatFusion_for_Breast_Cancer%5C1span%5CSKBR3_.html#read_11) | [4](../../../../J:%5CTophatFusion_for_Breast_Cancer%5C1span%5CSKBR3_.html#pair_11) | 1 |
| SK-200 | TATDN1 | chr8 | 125551167 | GSDMB | chr17 | 38066175 | [2](../../../../J:%5CTophatFusion_for_Breast_Cancer%5C1span%5CSKBR3_.html#read_4) | [14](../../../../J:%5CTophatFusion_for_Breast_Cancer%5C1span%5CSKBR3_.html#pair_4) | 9 |
| [SK-200](../../../../J:%5CTophatFusion_for_Breast_Cancer%5C1span%5CSKBR3_.html#fusion_11) | TATDN1 | chr8 | 125551262 | GSDMB | chr17 | 38062234 | [4](../../../../J:%5CTophatFusion_for_Breast_Cancer%5C1span%5CSKBR3_.html#read_2) | [14](../../../../J:%5CTophatFusion_for_Breast_Cancer%5C1span%5CSKBR3_.html#pair_2) | 21 |
| [SK-200](../../../../J:%5CTophatFusion_for_Breast_Cancer%5C1span%5CSKBR3_.html#fusion_11) | TATDN1 | chr8 | 125551264 | GSDMB | chr17 | 38062522 | [6](../../../../J:%5CTophatFusion_for_Breast_Cancer%5C1span%5CSKBR3_.html#read_3) | [14](../../../../J:%5CTophatFusion_for_Breast_Cancer%5C1span%5CSKBR3_.html#pair_3) | 18 |
| [SK-200](../../../../J:%5CTophatFusion_for_Breast_Cancer%5C1span%5CSKBR3_.html#fusion_11) | TATDN1 | chr8 | 125551264 | GSDMB | chr17 | 38066175 | [20](../../../../J:%5CTophatFusion_for_Breast_Cancer%5C1span%5CSKBR3_.html#read_5) | [14](../../../../J:%5CTophatFusion_for_Breast_Cancer%5C1span%5CSKBR3_.html#pair_5) | 63 |
| [SK-200](../../../../J:%5CTophatFusion_for_Breast_Cancer%5C1span%5CSKBR3_.html#fusion_11) | RARA | chr17 | 38465535 | PKIA | chr8 | 79479713 | [1](../../../../J:%5CTophatFusion_for_Breast_Cancer%5C1span%5CSKBR3_.html#read_6) | [5](../../../../J:%5CTophatFusion_for_Breast_Cancer%5C1span%5CSKBR3_.html#pair_6) | 1 |
| [SK-200](../../../../J:%5CTophatFusion_for_Breast_Cancer%5C1span%5CSKBR3_.html#fusion_11) | RARA | chr17 | 38465535 | PKIA | chr8 | 79510590 | [1](../../../../J:%5CTophatFusion_for_Breast_Cancer%5C1span%5CSKBR3_.html#read_7) | [3](../../../../J:%5CTophatFusion_for_Breast_Cancer%5C1span%5CSKBR3_.html#pair_7) | 1 |
| [SK-200](../../../../J:%5CTophatFusion_for_Breast_Cancer%5C1span%5CSKBR3_.html#fusion_11) | ENSG00000233955 | chr2 | 105011781 | AHCY | chr20 | 32873439 | [3](../../../../J:%5CTophatFusion_for_Breast_Cancer%5C1span%5CSKBR3_.html#read_0) | [6](../../../../J:%5CTophatFusion_for_Breast_Cancer%5C1span%5CSKBR3_.html#pair_0) | 0 |
| [SK-200](../../../../J:%5CTophatFusion_for_Breast_Cancer%5C1span%5CSKBR3_.html#fusion_11) | PRDX5 | chr11 | 64087339 | ENSG00000242364 | chr3 | 73697738 | [2](../../../../J:%5CTophatFusion_for_Breast_Cancer%5C1span%5CSKBR3_.html#read_9) | [7](../../../../J:%5CTophatFusion_for_Breast_Cancer%5C1span%5CSKBR3_.html#pair_9) | 3 |
| [SK-200](../../../../J:%5CTophatFusion_for_Breast_Cancer%5C1span%5CSKBR3_.html#fusion_11) | RBFOX2 | chr22 | 36293472 | NDUFA9 | chr12 | 4794423 | [1](../../../../J:%5CTophatFusion_for_Breast_Cancer%5C1span%5CSKBR3_.html#read_1) | [18](../../../../J:%5CTophatFusion_for_Breast_Cancer%5C1span%5CSKBR3_.html#pair_1) | 1 |
| [SK-200](../../../../J:%5CTophatFusion_for_Breast_Cancer%5C1span%5CSKBR3_.html#fusion_11) | ARL2BP | chr16 | 57283758 | ENSG00000215486 | chr13 | 37368983 | [1](../../../../J:%5CTophatFusion_for_Breast_Cancer%5C1span%5CSKBR3_.html#read_8) | [2](../../../../J:%5CTophatFusion_for_Breast_Cancer%5C1span%5CSKBR3_.html#pair_8) | 0 |
| [SK-200](../../../../J:%5CTophatFusion_for_Breast_Cancer%5C1span%5CSKBR3_.html#fusion_11) | PRDX3 | chr10 | 120936530 | PRICKLE2 | chr3 | 64160238 | [4](../../../../J:%5CTophatFusion_for_Breast_Cancer%5C1span%5CSKBR3_.html#read_10) | [1](../../../../J:%5CTophatFusion_for_Breast_Cancer%5C1span%5CSKBR3_.html#pair_10) | 1 |
| [BT](../../../../J:%5CTophatFusion_for_Breast_Cancer%5C1span%5CBT474.html#fusion_8)-100 | TRPC4AP | chr20 | 33665850 | MRPL45 | chr17 | 36478006 | [2](../../../../J:%5CTophatFusion_for_Breast_Cancer%5C1span%5CBT474.html#read_8) | [5](../../../../J:%5CTophatFusion_for_Breast_Cancer%5C1span%5CBT474.html#pair_8) | 5 |
| [BT](../../../../J:%5CTophatFusion_for_Breast_Cancer%5C1span%5CBT474.html#fusion_8)-100 | ENSG00000141232 | chr17 | 48943418 | SYNRG | chr17 | 35880750 | [12](../../../../J:%5CTophatFusion_for_Breast_Cancer%5C1span%5CBT474.html#read_13) | [8](../../../../J:%5CTophatFusion_for_Breast_Cancer%5C1span%5CBT474.html#pair_13) | 10 |
| [BT](../../../../J:%5CTophatFusion_for_Breast_Cancer%5C1span%5CBT474.html#fusion_8)-100 | ACACA | chr17 | 35479452 | STAC2 | chr17 | 37374425 | [31](../../../../J:%5CTophatFusion_for_Breast_Cancer%5C1span%5CBT474.html#read_15) | [6](../../../../J:%5CTophatFusion_for_Breast_Cancer%5C1span%5CBT474.html#pair_15) | 6 |
| [BT](../../../../J:%5CTophatFusion_for_Breast_Cancer%5C1span%5CBT474.html#fusion_8)-100 | MED1 | chr17 | 37595419 | ENSG00000167107 | chr17 | 48548386 | [8](../../../../J:%5CTophatFusion_for_Breast_Cancer%5C1span%5CBT474.html#read_16) | [5](../../../../J:%5CTophatFusion_for_Breast_Cancer%5C1span%5CBT474.html#pair_16) | 3 |
| [BT](../../../../J:%5CTophatFusion_for_Breast_Cancer%5C1span%5CBT474.html#fusion_8)-100 | STX16 | chr20 | 57227142 | RAE1 | chr20 | 55929087 | [4](../../../../J:%5CTophatFusion_for_Breast_Cancer%5C1span%5CBT474.html#read_29) | [8](../../../../J:%5CTophatFusion_for_Breast_Cancer%5C1span%5CBT474.html#pair_29) | 0 |
| [BT](../../../../J:%5CTophatFusion_for_Breast_Cancer%5C1span%5CBT474.html#fusion_8)-100 | ENSG00000150672 | chr11 | 85195025 | HFM1 | chr1 | 91853144 | [2](../../../../J:%5CTophatFusion_for_Breast_Cancer%5C1span%5CBT474.html#read_39) | [7](../../../../J:%5CTophatFusion_for_Breast_Cancer%5C1span%5CBT474.html#pair_39) | 2 |
| [BT](../../../../J:%5CTophatFusion_for_Breast_Cancer%5C1span%5CBT474.html#fusion_8)-100 | ZMYND8 | chr20 | 45852972 | CEP250 | chr20 | 34078459 | [6](../../../../J:%5CTophatFusion_for_Breast_Cancer%5C1span%5CBT474.html#read_9) | [11](../../../../J:%5CTophatFusion_for_Breast_Cancer%5C1span%5CBT474.html#pair_9) | 10 |
| [BT](../../../../J:%5CTophatFusion_for_Breast_Cancer%5C1span%5CBT474.html#fusion_8)-100 | ENSG00000153207 | chr1 | 247094879 | NAAA | chr4 | 76846963 | [6](../../../../J:%5CTophatFusion_for_Breast_Cancer%5C1span%5CBT474.html#read_36) | [9](../../../../J:%5CTophatFusion_for_Breast_Cancer%5C1span%5CBT474.html#pair_36) | 9 |
| [BT](../../../../J:%5CTophatFusion_for_Breast_Cancer%5C1span%5CBT474.html#fusion_8)-100 | MED13 | chr17 | 60129899 | BCAS3 | chr17 | 59469335 | [2](../../../../J:%5CTophatFusion_for_Breast_Cancer%5C1span%5CBT474.html#read_31) | [6](../../../../J:%5CTophatFusion_for_Breast_Cancer%5C1span%5CBT474.html#pair_31) | 1 |
| [BT](../../../../J:%5CTophatFusion_for_Breast_Cancer%5C1span%5CBT474.html#fusion_8)-100 | HFM1 | chr1 | 91853140 | ENSG00000198744 | chr1 | 570103 | [5](../../../../J:%5CTophatFusion_for_Breast_Cancer%5C1span%5CBT474.html#read_38) | [23](../../../../J:%5CTophatFusion_for_Breast_Cancer%5C1span%5CBT474.html#pair_38) | 1 |
| [BT](../../../../J:%5CTophatFusion_for_Breast_Cancer%5C1span%5CBT474.html#fusion_8)-100 | VAPB | chr20 | 56964572 | IKZF3 | chr17 | 37934019 | [9](../../../../J:%5CTophatFusion_for_Breast_Cancer%5C1span%5CBT474.html#read_28) | [10](../../../../J:%5CTophatFusion_for_Breast_Cancer%5C1span%5CBT474.html#pair_28) | 13 |
| [BT](../../../../J:%5CTophatFusion_for_Breast_Cancer%5C1span%5CBT474.html#fusion_8)-100 | ENSG00000251948 | chr19 | 24184149 | SLCO5A1 | chr8 | 70602608 | [1](../../../../J:%5CTophatFusion_for_Breast_Cancer%5C1span%5CBT474.html#read_3) | [3](../../../../J:%5CTophatFusion_for_Breast_Cancer%5C1span%5CBT474.html#pair_3) | 0 |
| [BT](../../../../J:%5CTophatFusion_for_Breast_Cancer%5C1span%5CBT474.html#fusion_8)-100 | PRDX5 | chr11 | 64087339 | ENSG00000242364 | chr3 | 73697738 | [51](../../../../J:%5CTophatFusion_for_Breast_Cancer%5C1span%5CBT474.html#read_32) | [169](../../../../J:%5CTophatFusion_for_Breast_Cancer%5C1span%5CBT474.html#pair_32) | 37 |
| [BT](../../../../J:%5CTophatFusion_for_Breast_Cancer%5C1span%5CBT474.html#fusion_8)-100 | NASP | chr1 | 46070687 | ENSG00000254777 | chr8 | 61852322 | [5](../../../../J:%5CTophatFusion_for_Breast_Cancer%5C1span%5CBT474.html#read_22) | [28](../../../../J:%5CTophatFusion_for_Breast_Cancer%5C1span%5CBT474.html#pair_22) | 1 |
| [BT](../../../../J:%5CTophatFusion_for_Breast_Cancer%5C1span%5CBT474.html#fusion_8)-100 | LDHA | chr11 | 18425239 | ENSG00000248527 | chr1 | 569473 | [2](../../../../J:%5CTophatFusion_for_Breast_Cancer%5C1span%5CBT474.html#read_2) | [3](../../../../J:%5CTophatFusion_for_Breast_Cancer%5C1span%5CBT474.html#pair_2) | 1 |
| [BT](../../../../J:%5CTophatFusion_for_Breast_Cancer%5C1span%5CBT474.html#fusion_8)-100 | WBSCR17 | chr7 | 70958325 | FBXL20 | chr17 | 37557612 | [1](../../../../J:%5CTophatFusion_for_Breast_Cancer%5C1span%5CBT474.html#read_34) | [2](../../../../J:%5CTophatFusion_for_Breast_Cancer%5C1span%5CBT474.html#pair_34) | 1 |
| [BT](../../../../J:%5CTophatFusion_for_Breast_Cancer%5C1span%5CBT474.html#fusion_8)-100 | GLB1 | chr3 | 33055545 | CMTM7 | chr3 | 32483333 | [1](../../../../J:%5CTophatFusion_for_Breast_Cancer%5C1span%5CBT474.html#read_7) | [2](../../../../J:%5CTophatFusion_for_Breast_Cancer%5C1span%5CBT474.html#pair_7) | 0 |
| [BT](../../../../J:%5CTophatFusion_for_Breast_Cancer%5C1span%5CBT474.html#fusion_8)-100 | STARD3 | chr17 | 37793479 | DOK5 | chr20 | 53259992 | [1](../../../../J:%5CTophatFusion_for_Breast_Cancer%5C1span%5CBT474.html#read_18) | [3](../../../../J:%5CTophatFusion_for_Breast_Cancer%5C1span%5CBT474.html#pair_18) | 3 |
| [BT](../../../../J:%5CTophatFusion_for_Breast_Cancer%5C1span%5CBT474.html#fusion_8)-100 | RAB22A | chr20 | 56886176 | MYO9B | chr19 | 17256205 | [6](../../../../J:%5CTophatFusion_for_Breast_Cancer%5C1span%5CBT474.html#read_27) | [1](../../../../J:%5CTophatFusion_for_Breast_Cancer%5C1span%5CBT474.html#pair_27) | 10 |
| [BT](../../../../J:%5CTophatFusion_for_Breast_Cancer%5C1span%5CBT474.html#fusion_8)-100 | ENSG00000150687 | chr11 | 86532805 | MOBKL3 | chr2 | 198380870 | [1](../../../../J:%5CTophatFusion_for_Breast_Cancer%5C1span%5CBT474.html#read_37) | [6](../../../../J:%5CTophatFusion_for_Breast_Cancer%5C1span%5CBT474.html#pair_37) | 3 |
| [BT](../../../../J:%5CTophatFusion_for_Breast_Cancer%5C1span%5CBT474.html#fusion_8)-100 | COPZ1 | chr12 | 54741832 | ENSG00000232155 | chrX | 153834114 | [2](../../../../J:%5CTophatFusion_for_Breast_Cancer%5C1span%5CBT474.html#read_25) | [5](../../../../J:%5CTophatFusion_for_Breast_Cancer%5C1span%5CBT474.html#pair_25) | 4 |
| [BT](../../../../J:%5CTophatFusion_for_Breast_Cancer%5C1span%5CBT474.html#fusion_8)-100 | MED1 | chr17 | 37607288 | STXBP4 | chr17 | 53218672 | [6](../../../../J:%5CTophatFusion_for_Breast_Cancer%5C1span%5CBT474.html#read_17) | [2](../../../../J:%5CTophatFusion_for_Breast_Cancer%5C1span%5CBT474.html#pair_17) | 3 |
| [BT](../../../../J:%5CTophatFusion_for_Breast_Cancer%5C1span%5CBT474.html#fusion_8)-100 | SKA2 | chr17 | 57232490 | MYO19 | chr17 | 34863349 | [1](../../../../J:%5CTophatFusion_for_Breast_Cancer%5C1span%5CBT474.html#read_10) | [2](../../../../J:%5CTophatFusion_for_Breast_Cancer%5C1span%5CBT474.html#pair_10) | 2 |
| [BT](../../../../J:%5CTophatFusion_for_Breast_Cancer%5C1span%5CBT474.html#fusion_8)-100 | SKA2 | chr17 | 57232490 | MYO19 | chr17 | 34863761 | [3](../../../../J:%5CTophatFusion_for_Breast_Cancer%5C1span%5CBT474.html#read_11) | [2](../../../../J:%5CTophatFusion_for_Breast_Cancer%5C1span%5CBT474.html#pair_11) | 5 |
| [BT](../../../../J:%5CTophatFusion_for_Breast_Cancer%5C1span%5CBT474.html#fusion_8)-100 | DIDO1 | chr20 | 61569147 | TTI1 | chr20 | 36634798 | [1](../../../../J:%5CTophatFusion_for_Breast_Cancer%5C1span%5CBT474.html#read_14) | [3](../../../../J:%5CTophatFusion_for_Breast_Cancer%5C1span%5CBT474.html#pair_14) | 1 |
| [BT](../../../../J:%5CTophatFusion_for_Breast_Cancer%5C1span%5CBT474.html#fusion_8)-100 | MLL3 | chr7 | 152064345 | ENSG00000226278 | chr7 | 55840870 | [1](../../../../J:%5CTophatFusion_for_Breast_Cancer%5C1span%5CBT474.html#read_45) | [8](../../../../J:%5CTophatFusion_for_Breast_Cancer%5C1span%5CBT474.html#pair_45) | 0 |
| [BT](../../../../J:%5CTophatFusion_for_Breast_Cancer%5C1span%5CBT474.html#fusion_8)-100 | GABRB3 | chr15 | 26790008 | RYR3 | chr15 | 33920673 | [2](../../../../J:%5CTophatFusion_for_Breast_Cancer%5C1span%5CBT474.html#read_4) | [1](../../../../J:%5CTophatFusion_for_Breast_Cancer%5C1span%5CBT474.html#pair_4) | 1 |
| [BT](../../../../J:%5CTophatFusion_for_Breast_Cancer%5C1span%5CBT474.html#fusion_8)-100 | RNF6 | chr13 | 26795971 | FOXO1 | chr13 | 41192773 | [1](../../../../J:%5CTophatFusion_for_Breast_Cancer%5C1span%5CBT474.html#read_5) | [5](../../../../J:%5CTophatFusion_for_Breast_Cancer%5C1span%5CBT474.html#pair_5) | 0 |
| [BT](../../../../J:%5CTophatFusion_for_Breast_Cancer%5C1span%5CBT474.html#fusion_8)-100 | GNAS | chr20 | 57484588 | ENSG00000248527 | chr1 | 569609 | [1](../../../../J:%5CTophatFusion_for_Breast_Cancer%5C1span%5CBT474.html#read_30) | [2](../../../../J:%5CTophatFusion_for_Breast_Cancer%5C1span%5CBT474.html#pair_30) | 0 |
| [BT](../../../../J:%5CTophatFusion_for_Breast_Cancer%5C1span%5CBT474.html#fusion_8)-100 | PKP4 | chr2 | 159440783 | ENSG00000240409 | chr1 | 569004 | [1](../../../../J:%5CTophatFusion_for_Breast_Cancer%5C1span%5CBT474.html#read_46) | [3](../../../../J:%5CTophatFusion_for_Breast_Cancer%5C1span%5CBT474.html#pair_46) | 0 |
| [BT](../../../../J:%5CTophatFusion_for_Breast_Cancer%5C1span%5CBT474.html#fusion_8)-100 | ENSG00000229344 | chr1 | 568361 | RYR2 | chr1 | 237766339 | [1](../../../../J:%5CTophatFusion_for_Breast_Cancer%5C1span%5CBT474.html#read_0) | [2](../../../../J:%5CTophatFusion_for_Breast_Cancer%5C1span%5CBT474.html#pair_0) | 0 |
| [BT](../../../../J:%5CTophatFusion_for_Breast_Cancer%5C1span%5CBT474.html#fusion_8)-100 | ACTN4 | chr19 | 39207954 | ACTN1 | chr14 | 69357003 | [4](../../../../J:%5CTophatFusion_for_Breast_Cancer%5C1span%5CBT474.html#read_21) | [4](../../../../J:%5CTophatFusion_for_Breast_Cancer%5C1span%5CBT474.html#pair_21) | 4 |
| [BT](../../../../J:%5CTophatFusion_for_Breast_Cancer%5C1span%5CBT474.html#fusion_8)-100 | ACTN1 | chr14 | 69360373 | ACTN4 | chr19 | 39207723 | [3](../../../../J:%5CTophatFusion_for_Breast_Cancer%5C1span%5CBT474.html#read_20) | [6](../../../../J:%5CTophatFusion_for_Breast_Cancer%5C1span%5CBT474.html#pair_20) | 1 |
| [BT](../../../../J:%5CTophatFusion_for_Breast_Cancer%5C1span%5CBT474.html#fusion_8)-100 | ANKRD30BL | chr2 | 133012085 | ENSG00000237973 | chr1 | 567103 | [2](../../../../J:%5CTophatFusion_for_Breast_Cancer%5C1span%5CBT474.html#read_42) | [2](../../../../J:%5CTophatFusion_for_Breast_Cancer%5C1span%5CBT474.html#pair_42) | 0 |
| [BT](../../../../J:%5CTophatFusion_for_Breast_Cancer%5C1span%5CBT474.html#fusion_8)-100 | ENSG00000235485 | chr22 | 34985972 | ACO2 | chr22 | 41918962 | [1](../../../../J:%5CTophatFusion_for_Breast_Cancer%5C1span%5CBT474.html#read_12) | [22](../../../../J:%5CTophatFusion_for_Breast_Cancer%5C1span%5CBT474.html#pair_12) | 0 |
| [BT](../../../../J:%5CTophatFusion_for_Breast_Cancer%5C1span%5CBT474.html#fusion_8)-100 | WBP11 | chr12 | 14953690 | WBP11P1 | chr18 | 30091903 | [2](../../../../J:%5CTophatFusion_for_Breast_Cancer%5C1span%5CBT474.html#read_6) | [43](../../../../J:%5CTophatFusion_for_Breast_Cancer%5C1span%5CBT474.html#pair_6) | 2 |
| [BT](../../../../J:%5CTophatFusion_for_Breast_Cancer%5C1span%5CBT474.html#fusion_8)-100 | RHEB | chr7 | 151199355 | ETF1 | chr5 | 137878393 | [2](../../../../J:%5CTophatFusion_for_Breast_Cancer%5C1span%5CBT474.html#read_44) | [1](../../../../J:%5CTophatFusion_for_Breast_Cancer%5C1span%5CBT474.html#pair_44) | 1 |
| [BT](../../../../J:%5CTophatFusion_for_Breast_Cancer%5C1span%5CBT474.html#fusion_8)-100 | RPS6KB1 | chr17 | 57970686 | SNF8 | chr17 | 47021335 | [32](../../../../J:%5CTophatFusion_for_Breast_Cancer%5C1span%5CBT474.html#read_23) | [11](../../../../J:%5CTophatFusion_for_Breast_Cancer%5C1span%5CBT474.html#pair_23) | 12 |
| [BT](../../../../J:%5CTophatFusion_for_Breast_Cancer%5C1span%5CBT474.html#fusion_8)-100 | RPS6KB1 | chr17 | 57971285 | SNF8 | chr17 | 47021335 | [1](../../../../J:%5CTophatFusion_for_Breast_Cancer%5C1span%5CBT474.html#read_24) | [11](../../../../J:%5CTophatFusion_for_Breast_Cancer%5C1span%5CBT474.html#pair_24) | 0 |
| [BT](../../../../J:%5CTophatFusion_for_Breast_Cancer%5C1span%5CBT474.html#fusion_8)-100 | DICER1 | chr14 | 95557544 | ENSG00000249894 | chr5 | 67151567 | [2](../../../../J:%5CTophatFusion_for_Breast_Cancer%5C1span%5CBT474.html#read_40) | [2](../../../../J:%5CTophatFusion_for_Breast_Cancer%5C1span%5CBT474.html#pair_40) | 2 |
| [BT](../../../../J:%5CTophatFusion_for_Breast_Cancer%5C1span%5CBT474.html#fusion_8)-100 | SEC14L1 | chr17 | 75187390 | ALKBH3 | chr11 | 43919323 | [1](../../../../J:%5CTophatFusion_for_Breast_Cancer%5C1span%5CBT474.html#read_35) | [3](../../../../J:%5CTophatFusion_for_Breast_Cancer%5C1span%5CBT474.html#pair_35) | 0 |
| [BT](../../../../J:%5CTophatFusion_for_Breast_Cancer%5C1span%5CBT474.html#fusion_8)-100 | MTIF2 | chr2 | 55470625 | ENSG00000228826 | chr1 | 121244943 | [1](../../../../J:%5CTophatFusion_for_Breast_Cancer%5C1span%5CBT474.html#read_26) | [8](../../../../J:%5CTophatFusion_for_Breast_Cancer%5C1span%5CBT474.html#pair_26) | 1 |
| [BT](../../../../J:%5CTophatFusion_for_Breast_Cancer%5C1span%5CBT474.html#fusion_8)-100 | ENSG00000239776 | chr12 | 127650981 | MTRNR2L2 | chr5 | 79946277 | [2](../../../../J:%5CTophatFusion_for_Breast_Cancer%5C1span%5CBT474.html#read_41) | [1](../../../../J:%5CTophatFusion_for_Breast_Cancer%5C1span%5CBT474.html#pair_41) | 0 |
| [BT](../../../../J:%5CTophatFusion_for_Breast_Cancer%5C1span%5CBT474.html#fusion_8)-100 | JAK2 | chr9 | 5112849 | TCF3 | chr19 | 1610500 | [1](../../../../J:%5CTophatFusion_for_Breast_Cancer%5C1span%5CBT474.html#read_1) | [29](../../../../J:%5CTophatFusion_for_Breast_Cancer%5C1span%5CBT474.html#pair_1) | 1 |
| [BT](../../../../J:%5CTophatFusion_for_Breast_Cancer%5C1span%5CBT474.html#fusion_8)-100 | SF3A3 | chr1 | 38455263 | ATRN | chr20 | 3471553 | [10](../../../../J:%5CTophatFusion_for_Breast_Cancer%5C1span%5CBT474.html#read_19) | [20](../../../../J:%5CTophatFusion_for_Breast_Cancer%5C1span%5CBT474.html#pair_19) | 0 |
| [BT](../../../../J:%5CTophatFusion_for_Breast_Cancer%5C1span%5CBT474.html#fusion_8)-100 | SDCCAG3 | chr9 | 139304541 | ENSG00000181101 | chr1 | 175014777 | [4](../../../../J:%5CTophatFusion_for_Breast_Cancer%5C1span%5CBT474.html#read_47) | [24](../../../../J:%5CTophatFusion_for_Breast_Cancer%5C1span%5CBT474.html#pair_47) | 4 |
| [BT](../../../../J:%5CTophatFusion_for_Breast_Cancer%5C1span%5CBT474.html#fusion_8)-100 | PCBD2 | chr5 | 134261791 | ANKRD30BL | chr2 | 133012976 | [2](../../../../J:%5CTophatFusion_for_Breast_Cancer%5C1span%5CBT474.html#read_43) | [1](../../../../J:%5CTophatFusion_for_Breast_Cancer%5C1span%5CBT474.html#pair_43) | 1 |
| [BT](../../../../J:%5CTophatFusion_for_Breast_Cancer%5C1span%5CBT474.html#fusion_8)-100 | ENSG00000226505 | chr2 | 70329650 | MRPL36 | chr5 | 1799907 | [8](../../../../J:%5CTophatFusion_for_Breast_Cancer%5C1span%5CBT474.html#read_33) | [19](../../../../J:%5CTophatFusion_for_Breast_Cancer%5C1span%5CBT474.html#pair_33) | 3 |
| [BT-2](../../../../J:%5CTophatFusion_for_Breast_Cancer%5C1span%5CBT474-2.html#fusion_8)00 | ENSG00000141232 | chr17 | 48943418 | SYNRG | chr17 | 35880750 | [14](../../../../J:%5CTophatFusion_for_Breast_Cancer%5C1span%5CBT474-2.html#read_8) | [14](../../../../J:%5CTophatFusion_for_Breast_Cancer%5C1span%5CBT474-2.html#pair_8) | 16 |
| [BT-2](../../../../J:%5CTophatFusion_for_Breast_Cancer%5C1span%5CBT474-2.html#fusion_8)00 | ACACA | chr17 | 35479452 | STAC2 | chr17 | 37374425 | [25](../../../../J:%5CTophatFusion_for_Breast_Cancer%5C1span%5CBT474-2.html#read_10) | [38](../../../../J:%5CTophatFusion_for_Breast_Cancer%5C1span%5CBT474-2.html#pair_10) | 8 |
| [BT-2](../../../../J:%5CTophatFusion_for_Breast_Cancer%5C1span%5CBT474-2.html#fusion_8)00 | THRA | chr17 | 38243102 | SKAP1 | chr17 | 46384689 | [11](../../../../J:%5CTophatFusion_for_Breast_Cancer%5C1span%5CBT474-2.html#read_12) | [11](../../../../J:%5CTophatFusion_for_Breast_Cancer%5C1span%5CBT474-2.html#pair_12) | 10 |
| [BT-2](../../../../J:%5CTophatFusion_for_Breast_Cancer%5C1span%5CBT474-2.html#fusion_8)00 | THRA | chr17 | 38243103 | SKAP1 | chr17 | 46371706 | [35](../../../../J:%5CTophatFusion_for_Breast_Cancer%5C1span%5CBT474-2.html#read_13) | [41](../../../../J:%5CTophatFusion_for_Breast_Cancer%5C1span%5CBT474-2.html#pair_13) | 28 |
| [BT-2](../../../../J:%5CTophatFusion_for_Breast_Cancer%5C1span%5CBT474-2.html#fusion_8)00 | TRPC4AP | chr20 | 33665850 | MRPL45 | chr17 | 36476499 | [2](../../../../J:%5CTophatFusion_for_Breast_Cancer%5C1span%5CBT474-2.html#read_5) | [2](../../../../J:%5CTophatFusion_for_Breast_Cancer%5C1span%5CBT474-2.html#pair_5) | 2 |
| [BT-2](../../../../J:%5CTophatFusion_for_Breast_Cancer%5C1span%5CBT474-2.html#fusion_8)00 | VAPB | chr20 | 56964570 | IKZF3 | chr17 | 37944628 | [1](../../../../J:%5CTophatFusion_for_Breast_Cancer%5C1span%5CBT474-2.html#read_18) | [3](../../../../J:%5CTophatFusion_for_Breast_Cancer%5C1span%5CBT474-2.html#pair_18) | 2 |
| [BT-2](../../../../J:%5CTophatFusion_for_Breast_Cancer%5C1span%5CBT474-2.html#fusion_8)00 | VAPB | chr20 | 56964572 | IKZF3 | chr17 | 37934019 | [10](../../../../J:%5CTophatFusion_for_Breast_Cancer%5C1span%5CBT474-2.html#read_19) | [17](../../../../J:%5CTophatFusion_for_Breast_Cancer%5C1span%5CBT474-2.html#pair_19) | 13 |
| [BT-2](../../../../J:%5CTophatFusion_for_Breast_Cancer%5C1span%5CBT474-2.html#fusion_8)00 | VAPB | chr20 | 56964574 | IKZF3 | chr17 | 37922743 | [4](../../../../J:%5CTophatFusion_for_Breast_Cancer%5C1span%5CBT474-2.html#read_20) | [9](../../../../J:%5CTophatFusion_for_Breast_Cancer%5C1span%5CBT474-2.html#pair_20) | 5 |
| [BT-2](../../../../J:%5CTophatFusion_for_Breast_Cancer%5C1span%5CBT474-2.html#fusion_8)00 | ENSG00000153207 | chr1 | 247094879 | NAAA | chr4 | 76846963 | [4](../../../../J:%5CTophatFusion_for_Breast_Cancer%5C1span%5CBT474-2.html#read_23) | [20](../../../../J:%5CTophatFusion_for_Breast_Cancer%5C1span%5CBT474-2.html#pair_23) | 4 |
| [BT-2](../../../../J:%5CTophatFusion_for_Breast_Cancer%5C1span%5CBT474-2.html#fusion_8)00 | ENSG00000248530 | chr3 | 131245709 | BCL2L12 | chr19 | 50172276 | [2](../../../../J:%5CTophatFusion_for_Breast_Cancer%5C1span%5CBT474-2.html#read_26) | [3](../../../../J:%5CTophatFusion_for_Breast_Cancer%5C1span%5CBT474-2.html#pair_26) | 4 |
| [BT-2](../../../../J:%5CTophatFusion_for_Breast_Cancer%5C1span%5CBT474-2.html#fusion_8)00 | HNRNPA2B1 | chr7 | 26236088 | ENSG00000248527 | chr1 | 569494 | [1](../../../../J:%5CTophatFusion_for_Breast_Cancer%5C1span%5CBT474-2.html#read_1) | [9](../../../../J:%5CTophatFusion_for_Breast_Cancer%5C1span%5CBT474-2.html#pair_1) | 0 |
| [BT-2](../../../../J:%5CTophatFusion_for_Breast_Cancer%5C1span%5CBT474-2.html#fusion_8)00 | MED13 | chr17 | 60129899 | BCAS3 | chr17 | 59469335 | [1](../../../../J:%5CTophatFusion_for_Breast_Cancer%5C1span%5CBT474-2.html#read_21) | [2](../../../../J:%5CTophatFusion_for_Breast_Cancer%5C1span%5CBT474-2.html#pair_21) | 2 |
| [BT-2](../../../../J:%5CTophatFusion_for_Breast_Cancer%5C1span%5CBT474-2.html#fusion_8)00 | SKA2 | chr17 | 57232490 | MYO19 | chr17 | 34863349 | [4](../../../../J:%5CTophatFusion_for_Breast_Cancer%5C1span%5CBT474-2.html#read_6) | [3](../../../../J:%5CTophatFusion_for_Breast_Cancer%5C1span%5CBT474-2.html#pair_6) | 5 |
| [BT-2](../../../../J:%5CTophatFusion_for_Breast_Cancer%5C1span%5CBT474-2.html#fusion_8)00 | SKA2 | chr17 | 57232490 | MYO19 | chr17 | 34863761 | [3](../../../../J:%5CTophatFusion_for_Breast_Cancer%5C1span%5CBT474-2.html#read_7) | [3](../../../../J:%5CTophatFusion_for_Breast_Cancer%5C1span%5CBT474-2.html#pair_7) | 4 |
| [BT-2](../../../../J:%5CTophatFusion_for_Breast_Cancer%5C1span%5CBT474-2.html#fusion_8)00 | UNC45B | chr17 | 33478354 | ENSG00000225630 | chr1 | 565857 | [1](../../../../J:%5CTophatFusion_for_Breast_Cancer%5C1span%5CBT474-2.html#read_4) | [14](../../../../J:%5CTophatFusion_for_Breast_Cancer%5C1span%5CBT474-2.html#pair_4) | 0 |
| [BT-2](../../../../J:%5CTophatFusion_for_Breast_Cancer%5C1span%5CBT474-2.html#fusion_8)00 | GLB1 | chr3 | 33055545 | CMTM7 | chr3 | 32483333 | [1](../../../../J:%5CTophatFusion_for_Breast_Cancer%5C1span%5CBT474-2.html#read_3) | [4](../../../../J:%5CTophatFusion_for_Breast_Cancer%5C1span%5CBT474-2.html#pair_3) | 0 |
| [BT-2](../../../../J:%5CTophatFusion_for_Breast_Cancer%5C1span%5CBT474-2.html#fusion_8)00 | ENSG00000239776 | chr12 | 127650883 | PPEF2 | chr4 | 76807305 | [1](../../../../J:%5CTophatFusion_for_Breast_Cancer%5C1span%5CBT474-2.html#read_25) | [5](../../../../J:%5CTophatFusion_for_Breast_Cancer%5C1span%5CBT474-2.html#pair_25) | 0 |
| [BT-2](../../../../J:%5CTophatFusion_for_Breast_Cancer%5C1span%5CBT474-2.html#fusion_8)00 | MED1 | chr17 | 37607288 | STXBP4 | chr17 | 53218672 | [7](../../../../J:%5CTophatFusion_for_Breast_Cancer%5C1span%5CBT474-2.html#read_11) | [1](../../../../J:%5CTophatFusion_for_Breast_Cancer%5C1span%5CBT474-2.html#pair_11) | 5 |
| [BT-2](../../../../J:%5CTophatFusion_for_Breast_Cancer%5C1span%5CBT474-2.html#fusion_8)00 | PRDX5 | chr11 | 64087339 | ENSG00000242364 | chr3 | 73697738 | [3](../../../../J:%5CTophatFusion_for_Breast_Cancer%5C1span%5CBT474-2.html#read_22) | [51](../../../../J:%5CTophatFusion_for_Breast_Cancer%5C1span%5CBT474-2.html#pair_22) | 19 |
| [BT-2](../../../../J:%5CTophatFusion_for_Breast_Cancer%5C1span%5CBT474-2.html#fusion_8)00 | PCBD2 | chr5 | 134262391 | CD63 | chr12 | 56119666 | [1](../../../../J:%5CTophatFusion_for_Breast_Cancer%5C1span%5CBT474-2.html#read_16) | [2](../../../../J:%5CTophatFusion_for_Breast_Cancer%5C1span%5CBT474-2.html#pair_16) | 1 |
| [BT-2](../../../../J:%5CTophatFusion_for_Breast_Cancer%5C1span%5CBT474-2.html#fusion_8)00 | PCBD2 | chr5 | 134263223 | CD63 | chr12 | 56120569 | [1](../../../../J:%5CTophatFusion_for_Breast_Cancer%5C1span%5CBT474-2.html#read_17) | [2](../../../../J:%5CTophatFusion_for_Breast_Cancer%5C1span%5CBT474-2.html#pair_17) | 1 |
| [BT-2](../../../../J:%5CTophatFusion_for_Breast_Cancer%5C1span%5CBT474-2.html#fusion_8)00 | RPS19 | chr19 | 42373782 | ENSG00000237973 | chr1 | 566916 | [2](../../../../J:%5CTophatFusion_for_Breast_Cancer%5C1span%5CBT474-2.html#read_14) | [2](../../../../J:%5CTophatFusion_for_Breast_Cancer%5C1span%5CBT474-2.html#pair_14) | 0 |
| [BT-2](../../../../J:%5CTophatFusion_for_Breast_Cancer%5C1span%5CBT474-2.html#fusion_8)00 | RPL23 | chr17 | 37009355 | ENSG00000225630 | chr1 | 565697 | [2](../../../../J:%5CTophatFusion_for_Breast_Cancer%5C1span%5CBT474-2.html#read_9) | [8](../../../../J:%5CTophatFusion_for_Breast_Cancer%5C1span%5CBT474-2.html#pair_9) | 1 |
| [BT-2](../../../../J:%5CTophatFusion_for_Breast_Cancer%5C1span%5CBT474-2.html#fusion_8)00 | RPS6KB1 | chr17 | 57970684 | SNF8 | chr17 | 47021337 | [17](../../../../J:%5CTophatFusion_for_Breast_Cancer%5C1span%5CBT474-2.html#read_15) | [21](../../../../J:%5CTophatFusion_for_Breast_Cancer%5C1span%5CBT474-2.html#pair_15) | 12 |
| [BT-2](../../../../J:%5CTophatFusion_for_Breast_Cancer%5C1span%5CBT474-2.html#fusion_8)00 | PEX19 | chr1 | 160253425 | ENSG00000225630 | chr1 | 565873 | [1](../../../../J:%5CTophatFusion_for_Breast_Cancer%5C1span%5CBT474-2.html#read_28) | [4](../../../../J:%5CTophatFusion_for_Breast_Cancer%5C1span%5CBT474-2.html#pair_28) | 0 |
| [BT-2](../../../../J:%5CTophatFusion_for_Breast_Cancer%5C1span%5CBT474-2.html#fusion_8)00 | TBX3 | chr12 | 115108612 | ENSG00000237973 | chr1 | 566965 | [1](../../../../J:%5CTophatFusion_for_Breast_Cancer%5C1span%5CBT474-2.html#read_24) | [2](../../../../J:%5CTophatFusion_for_Breast_Cancer%5C1span%5CBT474-2.html#pair_24) | 0 |
| [BT-2](../../../../J:%5CTophatFusion_for_Breast_Cancer%5C1span%5CBT474-2.html#fusion_8)00 | JAK2 | chr9 | 5112849 | TCF3 | chr19 | 1610500 | [1](../../../../J:%5CTophatFusion_for_Breast_Cancer%5C1span%5CBT474-2.html#read_0) | [17](../../../../J:%5CTophatFusion_for_Breast_Cancer%5C1span%5CBT474-2.html#pair_0) | 1 |
| [BT-2](../../../../J:%5CTophatFusion_for_Breast_Cancer%5C1span%5CBT474-2.html#fusion_8)00 | TUBB2C | chr9 | 140136278 | TUBB | chr6 | 30690692 | [2](../../../../J:%5CTophatFusion_for_Breast_Cancer%5C1span%5CBT474-2.html#read_27) | [1](../../../../J:%5CTophatFusion_for_Breast_Cancer%5C1span%5CBT474-2.html#pair_27) | 0 |
| [BT-2](../../../../J:%5CTophatFusion_for_Breast_Cancer%5C1span%5CBT474-2.html#fusion_8)00 | XBP1 | chr22 | 29192181 | REEP5 | chr5 | 112221156 | [3](../../../../J:%5CTophatFusion_for_Breast_Cancer%5C1span%5CBT474-2.html#read_2) | [1](../../../../J:%5CTophatFusion_for_Breast_Cancer%5C1span%5CBT474-2.html#pair_2) | 1 |
| [KPL4](../../../../J:%5CTophatFusion_for_Breast_Cancer%5C1span%5CKPL4.html#fusion_0) | BSG | chr19 | 580779 | NFIX | chr19 | 13135832 | [9](../../../../J:%5CTophatFusion_for_Breast_Cancer%5C1span%5CKPL4.html#read_0) | [11](../../../../J:%5CTophatFusion_for_Breast_Cancer%5C1span%5CKPL4.html#pair_0) | 4 |
| [KPL4](../../../../J:%5CTophatFusion_for_Breast_Cancer%5C1span%5CKPL4.html#fusion_5) | MUC20 | chr3 | 195456606 | ENSG00000236833 | chr3 | 197391655 | [2](../../../../J:%5CTophatFusion_for_Breast_Cancer%5C1span%5CKPL4.html#read_5) | [4](../../../../J:%5CTophatFusion_for_Breast_Cancer%5C1span%5CKPL4.html#pair_5) | 2 |
| [KPL4](../../../../J:%5CTophatFusion_for_Breast_Cancer%5C1span%5CKPL4.html#fusion_1) | NASP | chr1 | 46070685 | ENSG00000254777 | chr8 | 61852324 | [1](../../../../J:%5CTophatFusion_for_Breast_Cancer%5C1span%5CKPL4.html#read_1) | [10](../../../../J:%5CTophatFusion_for_Breast_Cancer%5C1span%5CKPL4.html#pair_1) | 1 |
| [KPL4](../../../../J:%5CTophatFusion_for_Breast_Cancer%5C1span%5CKPL4.html#fusion_2) | COPZ1 | chr12 | 54741832 | ENSG00000232155 | chrX | 153834114 | [1](../../../../J:%5CTophatFusion_for_Breast_Cancer%5C1span%5CKPL4.html#read_2) | [2](../../../../J:%5CTophatFusion_for_Breast_Cancer%5C1span%5CKPL4.html#pair_2) | 1 |
| [KPL4](../../../../J:%5CTophatFusion_for_Breast_Cancer%5C1span%5CKPL4.html#fusion_4) | SEC14L1 | chr17 | 75187390 | ALKBH3 | chr11 | 43919323 | [1](../../../../J:%5CTophatFusion_for_Breast_Cancer%5C1span%5CKPL4.html#read_4) | [3](../../../../J:%5CTophatFusion_for_Breast_Cancer%5C1span%5CKPL4.html#pair_4) | 0 |
| [KPL4](../../../../J:%5CTophatFusion_for_Breast_Cancer%5C1span%5CKPL4.html#fusion_3) | PRDX5 | chr11 | 64087339 | ENSG00000242364 | chr3 | 73697738 | [1](../../../../J:%5CTophatFusion_for_Breast_Cancer%5C1span%5CKPL4.html#read_3) | [5](../../../../J:%5CTophatFusion_for_Breast_Cancer%5C1span%5CKPL4.html#pair_3) | 1 |
| [MCF7](../../../../J:%5CTophatFusion_for_Breast_Cancer%5C1span%5CMCF7.html#fusion_7) | BCAS4 | chr20 | 49411707 | BCAS3 | chr17 | 59430946 | [3](../../../../J:%5CTophatFusion_for_Breast_Cancer%5C1span%5CMCF7.html#read_7) | [1](../../../../J:%5CTophatFusion_for_Breast_Cancer%5C1span%5CMCF7.html#pair_7) | 3 |
| [MCF7](../../../../J:%5CTophatFusion_for_Breast_Cancer%5C1span%5CMCF7.html#fusion_8) | BCAS4 | chr20 | 49411707 | BCAS3 | chr17 | 59445685 | [33](../../../../J:%5CTophatFusion_for_Breast_Cancer%5C1span%5CMCF7.html#read_8) | [39](../../../../J:%5CTophatFusion_for_Breast_Cancer%5C1span%5CMCF7.html#pair_8) | 54 |
| [MCF7](../../../../J:%5CTophatFusion_for_Breast_Cancer%5C1span%5CMCF7.html#fusion_3) | ARFGEF2 | chr20 | 47538545 | SULF2 | chr20 | 46365686 | [14](../../../../J:%5CTophatFusion_for_Breast_Cancer%5C1span%5CMCF7.html#read_3) | [8](../../../../J:%5CTophatFusion_for_Breast_Cancer%5C1span%5CMCF7.html#pair_3) | 5 |
| [MCF7](../../../../J:%5CTophatFusion_for_Breast_Cancer%5C1span%5CMCF7.html#fusion_11) | ENSG00000224738 | chr17 | 57184949 | TMEM49 | chr17 | 57915653 | [5](../../../../J:%5CTophatFusion_for_Breast_Cancer%5C1span%5CMCF7.html#read_11) | [3](../../../../J:%5CTophatFusion_for_Breast_Cancer%5C1span%5CMCF7.html#pair_11) | 3 |
| [MCF7](../../../../J:%5CTophatFusion_for_Breast_Cancer%5C1span%5CMCF7.html#fusion_4) | SULF2 | chr20 | 46415145 | ENSG00000171940 | chr20 | 52210297 | [10](../../../../J:%5CTophatFusion_for_Breast_Cancer%5C1span%5CMCF7.html#read_4) | [12](../../../../J:%5CTophatFusion_for_Breast_Cancer%5C1span%5CMCF7.html#pair_4) | 14 |
| [MCF7](../../../../J:%5CTophatFusion_for_Breast_Cancer%5C1span%5CMCF7.html#fusion_5) | SULF2 | chr20 | 46415146 | ENSG00000171940 | chr20 | 52210647 | [8](../../../../J:%5CTophatFusion_for_Breast_Cancer%5C1span%5CMCF7.html#read_5) | [12](../../../../J:%5CTophatFusion_for_Breast_Cancer%5C1span%5CMCF7.html#pair_5) | 9 |
| [MCF7](../../../../J:%5CTophatFusion_for_Breast_Cancer%5C1span%5CMCF7.html#fusion_10) | COPZ1 | chr12 | 54741832 | ENSG00000232155 | chrX | 153834114 | [2](../../../../J:%5CTophatFusion_for_Breast_Cancer%5C1span%5CMCF7.html#read_10) | [4](../../../../J:%5CTophatFusion_for_Breast_Cancer%5C1span%5CMCF7.html#pair_10) | 2 |
| [MCF7](../../../../J:%5CTophatFusion_for_Breast_Cancer%5C1span%5CMCF7.html#fusion_6) | RBM10 | chrX | 47017169 | ENSG00000100605 | chr14 | 93542939 | [4](../../../../J:%5CTophatFusion_for_Breast_Cancer%5C1span%5CMCF7.html#read_6) | [1](../../../../J:%5CTophatFusion_for_Breast_Cancer%5C1span%5CMCF7.html#pair_6) | 5 |
| [MCF7](../../../../J:%5CTophatFusion_for_Breast_Cancer%5C1span%5CMCF7.html#fusion_1) | ACTN4 | chr19 | 39207954 | ACTN1 | chr14 | 69357003 | [4](../../../../J:%5CTophatFusion_for_Breast_Cancer%5C1span%5CMCF7.html#read_1) | [5](../../../../J:%5CTophatFusion_for_Breast_Cancer%5C1span%5CMCF7.html#pair_1) | 4 |
| [MCF7](../../../../J:%5CTophatFusion_for_Breast_Cancer%5C1span%5CMCF7.html#fusion_12) | PRDX5 | chr11 | 64087339 | ENSG00000242364 | chr3 | 73697738 | [4](../../../../J:%5CTophatFusion_for_Breast_Cancer%5C1span%5CMCF7.html#read_12) | [15](../../../../J:%5CTophatFusion_for_Breast_Cancer%5C1span%5CMCF7.html#pair_12) | 4 |
| [MCF7](../../../../J:%5CTophatFusion_for_Breast_Cancer%5C1span%5CMCF7.html#fusion_14) | ENSG00000216676 | chr6 | 27620413 | RPL8 | chr8 | 146015845 | [1](../../../../J:%5CTophatFusion_for_Breast_Cancer%5C1span%5CMCF7.html#read_14) | [18](../../../../J:%5CTophatFusion_for_Breast_Cancer%5C1span%5CMCF7.html#pair_14) | 1 |
| [MCF7](../../../../J:%5CTophatFusion_for_Breast_Cancer%5C1span%5CMCF7.html#fusion_0) | ACTN1 | chr14 | 69360373 | ACTN4 | chr19 | 39207723 | [1](../../../../J:%5CTophatFusion_for_Breast_Cancer%5C1span%5CMCF7.html#read_0) | [5](../../../../J:%5CTophatFusion_for_Breast_Cancer%5C1span%5CMCF7.html#pair_0) | 2 |
| [MCF7](../../../../J:%5CTophatFusion_for_Breast_Cancer%5C1span%5CMCF7.html#fusion_9) | AP2A1 | chr19 | 50302749 | AP2A2 | chr11 | 988549 | [3](../../../../J:%5CTophatFusion_for_Breast_Cancer%5C1span%5CMCF7.html#read_9) | [1](../../../../J:%5CTophatFusion_for_Breast_Cancer%5C1span%5CMCF7.html#pair_9) | 0 |
| [MCF7](../../../../J:%5CTophatFusion_for_Breast_Cancer%5C1span%5CMCF7.html#fusion_2) | NASP | chr1 | 46070685 | ENSG00000254777 | chr8 | 61852324 | [3](../../../../J:%5CTophatFusion_for_Breast_Cancer%5C1span%5CMCF7.html#read_2) | [7](../../../../J:%5CTophatFusion_for_Breast_Cancer%5C1span%5CMCF7.html#pair_2) | 2 |
| [MCF7](../../../../J:%5CTophatFusion_for_Breast_Cancer%5C1span%5CMCF7.html#fusion_13) | ENSG00000226505 | chr2 | 70329650 | MRPL36 | chr5 | 1799907 | [5](../../../../J:%5CTophatFusion_for_Breast_Cancer%5C1span%5CMCF7.html#read_13) | [11](../../../../J:%5CTophatFusion_for_Breast_Cancer%5C1span%5CMCF7.html#pair_13) | 1 |

SK-100, SK-200 is the data from SKBR3 cell line with reads length 100, 200 respectively

BT-100, BT-200 is the data from BT474 cell line with reads length 100,200 respectively
